# Multi-omics characterization of a rhizosphere-derived *Bacillus cereus* CBS-B5 strain reveals genomic stability, metabolic versatility, and biosafety-related genomic features for agricultural applications

**DOI:** 10.3389/fmicb.2026.1857756

**Published:** 2026-09-04

**Authors:** Al-Sayed Al-Soudy, Wessam M. Rslan, Bouchra Soulaimani, Omar Hajjaji, Haytham M. Abd-Elhalim, Badr Eddine Drissi, Mansour Sobeh, Rachid Daoud, Rachid Benhida, Morad M. Mokhtar

**Affiliations:** 1 Chemical and Biochemical Sciences-Green Process Engineering, College of Chemical Sciences and Engineering, Mohammed VI Polytechnic University, Benguerir, Morocco; 2 Agricultural Genetic Engineering Research Institute, Agricultural Research Center, Giza, Egypt; 3 Agrobiosciences Program, College of Agriculture and Environmental Sciences, University Mohammed VI Polytechnic, Ben Guerir, Morocco; 4 Institute of Chemistry, Nice UMR7272, Côte d'Azur University, French National Centre for Scientific Research (CNRS), Nice, France

**Keywords:** antimicrobial resistance, *Bacillus cereus*, genome sequencing, multi-omics integration, plant growth-promoting rhizobacteria, sugar beet rhizosphere, virulence factors

## Abstract

**Introduction:**

*Bacillus cereus* strains have potential plant growth-promoting properties but may harbor virulence and antimicrobial resistance (AMR) determinants. This study characterized the rhizosphere-derived *B. cereus* CBS-B5 strain to assess its functional potential and biosafety-related features.

**Methods:**

CBS-B5, isolated from sugar beet rhizosphere, was characterized using an integrated multi-omics approach combining phenotypic assays, whole-genome sequencing, phylogenomic and comparative genomic analyses, and metabolomic profiling.

**Results:**

CBS-B5 exhibited visible growth under elevated salinity conditions, demonstrated recovery following heat stress exposure, and strong biofilm formation, but no detectable phosphate solubilization. Whole-genome sequencing revealed a 5.02 Mb genome with 35% GC content, 100% completeness, and 0.03% contamination. Phylogenomic analysis placed CBS-B5 within the *B. cereus* group. Comparative genomic and functional analyses indicated genomic stability, metabolic versatility, stress-adaptation potential, and diverse biosynthetic gene clusters. Genome plasticity was supported by the presence of mobile genetic elements and horizontal gene transfer events affecting approximately 16% of the proteome. Metabolomic analysis confirmed active metabolic processes, including nitrogen recycling, osmoprotection, and transformation of plant-derived compounds under laboratory conditions. Although virulence-associated genes, including *nheABC, cytK*, and *inhA*, and β-hemolytic activity were detected, AMR and virulence determinants showed limited potential for horizontal dissemination. Similarly, AMR genes exhibited low mobility potential and minimal phenotypic resistance beyond intrinsic traits.

**Discussion:**

Overall, CBS-B5 combines genomic stability, metabolic flexibility, and ecological adaptability. From a One Health perspective, the genomic analyses suggest a limited potential for horizontal dissemination of antimicrobial resistance and virulence determinants. However, the presence of chromosomally encoded toxin-associated genes and β-hemolytic activity indicates that additional biosafety evaluation is required before agricultural application.

## 1 Introduction

The *Bacillus cereus* group (also referred to as *B. cereus* sensu lato) comprises a diverse lineage of Gram-positive, rod-shaped, endospore-forming bacteria widely distributed across environmental niches, particularly in soil and plant-associated ecosystems (Zeng et al., 2018). Members of this group exhibit remarkable ecological and metabolic versatility, ranging from saprophytic lifestyles in soil to symbiotic lifestyles near plant roots, as well as pathogenic interactions with insect and mammal hosts (Ceuppens et al., 2013; Ehling-Schulz et al., 2019). This dual ecological role makes *B. cereus* both a promising candidate for agricultural applications and a subject of biosafety concern.

The *B. cereus* group is also notable for producing a variety of valuable enzymes and metabolites (Nilegaonkar et al., 2007), degrading different types of pollutants, and promoting the growth of both plants and animals when used as probiotics (Guinebretière et al., 2013; Hong et al., 2005). Several *B. cereus* strains have been identified as plant growth–promoting rhizobacteria (PGPR), capable of enhancing plant growth through multiple mechanisms, including nutrient mobilization, phosphate solubilization, siderophore-mediated iron (Fe) acquisition, phytohormone production, and stress mitigation (Paterson et al., 2017; Grobelak et al., 2015; Garcia-Pausas and Paterson, 2011). These beneficial traits have supported the development of *B. cereus*-based biofertilizers and biocontrol agents, with demonstrated effectiveness in improving crop productivity under both controlled and field conditions (Ku et al., 2018; Sansinenea, 2019; Ali et al., 2021; Liu et al., 2023; Bakhshandeh et al., 2020). For example, *B. cereus* strain 905, isolated from the wheat rhizosphere, has been successfully applied as a biopesticide across large agricultural areas (approximately 3 million acres of wheat fields), highlighting its practical relevance for sustainable agriculture (Wang et al., 2007). The draft genome sequence of this strain has served as a valuable genetic resource for investigating *B. cereus*–plant interactions and identifying genes associated with plant growth promotion and biocontrol potential (Ding et al., 2016).

In addition to promoting plant growth, *B. cereus* strains contribute to environmental resilience by facilitating nutrient cycling and enhancing plant tolerance to abiotic stresses such as salinity, heat, and heavy metal toxicity (Andy et al., 2023; Jabeen et al., 2022; Sahile et al., 2021; Akhtar et al., 2021; Abou-Aly et al., 2021). These characteristics contribute to the ecological persistence of *B. cereus* under fluctuating environmental conditions and are particularly relevant for agricultural systems in arid and semi-arid regions. In addition, several studies have demonstrated that bacterial consortia containing *B. cereus* can promote the growth of crops and medicinal plants under controlled conditions (Sivasankari and Anandharaj, 2014; Elsayed et al., 2022; Chauhan et al., 2017; Hassan and Bano, 2016).

In light of the significance of the *B. cereus* group, numerous studies have focused on classifying and identifying isolates using genome-based approaches. According to the NCBI Microbial Genomes database (https://www.ncbi.nlm.nih.gov/datasets/genome/, accessed in March 2026), there are 3,505 complete genomes available for *B. cereus*.

Despite these advantages, some members of the *B. cereus* group harbor virulence factors and antimicrobial resistance (AMR) genes, including enterotoxins such as hemolysin BL (HBL), non-hemolytic enterotoxin (NHE), and cytotoxin K (CytK), which are associated with foodborne illnesses in humans and animals (Etikala et al., 2022; Dietrich et al., 2021). Therefore, distinguishing beneficial rhizosphere-associated strains from potentially harmful lineages remains a major challenge and highlights the importance of evaluating microbial candidates for agricultural use within a One Health framework that integrates environmental, plant, and human health considerations (Etikala et al., 2022; Dietrich et al., 2021).

Recent advances in high-throughput sequencing technologies have enabled detailed genomic and functional analyses of *B. cereus*, providing new insights into genome architecture and ecological adaptation. Comparative genomics has revealed that the *B. cereus* group possesses an open pangenome, reflecting extensive genome plasticity driven by horizontal gene transfer (HGT), mobile genetic elements (MGEs), and environmental adaptation (Kim et al., 2017; Koo et al., 2017; Bazinet, 2017). While core genes associated with plant interactions are generally conserved across strains, accessory genomes contribute to functional diversity and niche specialization (Zeng et al., 2018; Adeleke et al., 2021b).

Despite these advances, linking genomic potential with experimentally validated phenotypes and metabolic activity remains a major challenge. Relatively few studies have integrated genome-scale analyses with phenotypic characterization and metabolomic profiling to establish functional relevance under environmentally relevant conditions (Kim et al., 2017; Koo et al., 2017; Bazinet, 2017). Integrative multi-omics approaches provide a powerful framework for addressing this challenge by combining genomic, metabolomic, and phenotypic analyses to evaluate metabolic versatility, stress adaptation, and biosafety-related genomic features. Integrating genomic predictions with experimentally validated phenotypes is essential for improving our understanding of genotype–phenotype relationships in rhizosphere-associated bacteria.

In the present study, we isolated and characterized a rhizosphere-derived *B. cereus* strain (CBS-B5), from sugar beet (*Beta vulgaris* L.) rhizosphere soil. An integrated multi-omics approach combining phenotypic characterization, whole-genome sequencing, phylogenomic analysis, comparative genome analysis, and metabolomic profiling was employed to investigate the ecological adaptation, metabolic versatility, predicted plant growth-promoting potential, and biosafety-related genomic features of this strain. The study aimed to provide a comprehensive functional characterization of CBS-B5 and to establish a framework for future *in planta* validation and biosafety assessment.

## 2 Material and method

### 2.1 Bacterial isolation and morphological characterization

Rhizosphere soil samples were collected from a sugar beet (*Beta vulgaris* L.) field located in Giza, Egypt. Samples were obtained from the top 10–15 cm of soil surrounding the roots of healthy sugar beet plants. Approximately 100 g of soil was collected using a sterile spatula and stored in sterile polyethylene bags at 4 °C until processing, while glycerol stocks (20% v/v) were prepared and stored at –80 °C for long-term preservation.

For the isolation, 1 g of rhizosphere soil was suspended in 9 mL of sterile saline solution (0.85% NaCl) and vortexed for 1 min to obtain a homogeneous suspension. Serial tenfold dilutions (10^−1^ to 10^−6^) were prepared using sterile saline. Aliquots of 100 μL from appropriate dilutions were spread evenly onto nutrient agar (NA) plates. The inoculated plates were incubated aerobically at 32 °C for 24–48 h. Morphologically distinct colonies were selected and purified by repeated streaking on NA plates until pure cultures were obtained.

For morphological characterization, the CBS-B5 strain was examined macroscopically by colony morphology on NA plates after incubation at 32 °C for 24-48 h. Colony characteristics, including size, shape, margin, elevation, surface texture, pigmentation, and opacity, were recorded. To obtain microscopic images, morphological characterization was carried out using light microscopy following Gram staining. Bacterial smears were prepared from 24-h-old cultures, air-dried, and heat-fixed. Slides were stained using crystal violet as the primary stain, iodine as the mordant, alcohol for decolorization, and safranin as the counterstain. Stained preparations were examined under a light microscope using oil immersion (100 × magnification) to determine cell shape, arrangement, and Gram reaction. Endospore formation was assessed using the malachite green staining technique. Heat-fixed smears were stained with malachite green and gently heated for 5–10 min to allow dye penetration into endospores. Excess stain was removed by rinsing with distilled water, and slides were counterstained with safranin for 30–60 s. Microscopic examination was performed under oil immersion. Endospores appeared green or light blue, whereas vegetative cells appeared pink or red. Morphological characteristics observed at both macroscopic and microscopic levels were compared with standard taxonomic descriptions of *Bacillus cereus*.

### 2.2 Salt tolerance and heat stress recovery assays

The salt tolerance of the CBS-B5 culture was determined using the ability of the bacteria to grow and develop on a nutrient agar (NA) medium supplemented with different concentrations of NaCl. The NA plates were prepared with increasing concentrations of NaCl to represent 1% (0.172 M), 2.5% (0.43 M), 5.0% (0.86 M), 7.5% (1.29 M), 10.0% (1.72 M), and 11.62% (2 M). The NA medium without the addition of NaCl was used as the control. A fresh bacterial culture of strain CBS-B5 was streaked on the surface of the prepared NA plate. The inoculated plate was incubated at 30 °C for 24–48 h. After incubation, the bacterial growth was evaluated using visual observation. The strain was considered salt-resistant when visible growth was observed on nutrient agar supplemented with NaCl concentrations of 7.5% (w/v) or higher (Egamberdieva et al., 2015; Kushner, 2020; Ventosa et al., 2008).

The heat tolerance of the CBS-B5 was assessed based on its ability to recover and grow following exposure to thermal stress using a broth-based assay in tryptic soy broth (TSB). A fresh bacterial culture was grown and standardized to an optical density of approximately OD_600_ = 0.4. Aliquots of the standardized bacterial suspension (4 mL) were subjected to heat stress by incubation at different temperatures, including 30 °C (control), 40 °C, 50 °C, 60 °C, and 65 °C, for 30 min. Immediately after heat exposure, the samples were rapidly cooled by immersion in an ice-cold bath for 5 min to stop heat stress. Following the heat stress treatment, cultures were incubated at 30 °C overnight. After 24 h of incubation, bacterial growth was quantified by measuring the optical density at 600 nm (OD_600_) using a UV–visible spectrophotometer (Agilent 8453 UV–Visible Spectroscopy System). The strain was considered heat-tolerant when measurable growth was observed following exposure to elevated temperatures (≥50 °C) in culture broth. Recovery following heat stress was evaluated by comparing post-treatment growth values with those of the untreated control maintained at 30 °C.

### 2.3 Phosphate solubilization and biofilm formation assays

The ability of CBS-B5 to solubilize phosphate ions was assessed using Pikovskaya's agar medium (PVK agar). The PVK agar medium was prepared with the following composition (g L^−1^): yeast extract, 0.5; dextrose, 10.0; calcium phosphate, 5.0; ammonium sulfate, 0.5; potassium chloride, 0.2; magnesium sulfate, 0.1; manganese sulfate, 0.0001; ferrous sulfate, 0.0001; and agar, 20.0. The medium was sterilized by autoclaving at 121 °C for 15 min and poured into sterile Petri dishes. A fresh culture of CBS-B5 was spot-inoculated onto the surface of PVK agar plates using a sterile inoculating loop. The inoculated plates were incubated aerobically at 32 °C for 7 days. Following incubation, the plates were examined for the formation of clear halo zones surrounding bacterial colonies, indicating phosphate solubilization activity.

The biofilm-forming ability of CBS-B5 was evaluated using the crystal violet staining assay in 96-well polystyrene microtiter plates. Bacterial suspensions were prepared in TSB supplemented with 1% (w/v) glucose and adjusted to a 0.5 McFarland standard. Aliquots of 200 μL from each suspension were dispensed into individual wells. The experiment was performed in triplicate, with three wells containing uninoculated medium serving as negative controls. After incubation at 32 °C for 48 h, the supernatant was gently removed, and the wells were washed three times with sterile phosphate-buffered saline (PBS; pH 7.3) to remove non-adherent cells. The plates were air-dried and subsequently stained with 0.1% (w/v) aqueous crystal violet solution for 20 min. Excess stain was removed by washing the wells three times with PBS. The bound crystal violet was solubilized by adding 200 μL of 100% ethanol to each well. Absorbance was measured at 530 nm using a microplate reader, with plates briefly shaken prior to measurement to ensure uniform mixing. Biofilm formation was quantified by comparing the optical density of the test sample (OD_s_) with that of the negative control (OD_c_). The cut-off optical density (OD_c_) was defined as the arithmetic mean absorbance of the negative controls plus three times the standard deviation (OD_c_ = mean OD of negative control + 3 × SD). Biofilm production was classified as follows: no biofilm production (OD_s_ ≤ OD_c_), weak biofilm production (OD_c_ < OD_s_ ≤ 2OD_c_), moderate biofilm production (2OD_c_ < OD_s_ ≤ 4OD_c_), and strong biofilm production (OD_s_ > 4OD_c_) (Stepanović et al., 2007).

### 2.4 Hemolytic activity and antimicrobial susceptibility assays

The hemolytic activity of CBS-B5 was evaluated as a preliminary safety assessment following standard microbiological procedures (Cappuccino and Welsh, 2017). The strain was streaked onto 5% (v/v) blood agar plates and incubated at 32 °C for 48 h. After incubation, hemolytic activity was determined by visual inspection of the zones surrounding the colonies. Hemolysis was classified as α-hemolysis, indicated by a greenish discoloration due to partial lysis of red blood cells; β-hemolysis, characterized by a clear zone resulting from complete lysis; or γ-hemolysis, defined by the absence of visible changes in the medium. The hemolytic profile of strain CBS-B5 was recorded accordingly.

The antibiotic susceptibility of the CBS-B5 was assessed on Mueller-Hinton agar (MHA) using the disc diffusion method as described by Al-Muzahmi et al. (2023). Bacterial colonies from overnight pure cultures were suspended in normal saline (Fisher Chemical, Cramlington, UK) and adjusted to a turbidity equivalent to a 0.5 McFarland standard using a CrystalSpec nephelometer (BD Diagnostics, Sparks Glencoe, MD, USA), following the manufacturer's recommendations. After 15 minutes, the bacterial suspension was evenly spread onto a Mueller-Hinton agar (MHA, Oxoid, Hampshire, UK) surface plate and allowed to absorb for 1–2 min at room temperature. After an additional 15 minutes, the selected antibiotic disks (BioMérieux and Liofilchem, Germany) were placed on the inoculated MHA plates using sterile forceps. The plates were incubated at 32 °C for 18 h. The antibiotics tested against the CBS-B5 strain included chloramphenicol 30 μg, ceftazidime 30 μg, azithromycin 15 μg, tetracycline 30 μg, penicillin 10 μg, and mitomycin 30 μg. The diameter of the zones of growth inhibition around each antibiotic disc was measured in millimeters to evaluate the antibacterial effect and to determine susceptibility. The results were interpreted in accordance with the Clinical and Laboratory Standards Institute (CLSI) guidelines. All experiments were performed in triplicate to ensure reproducibility.

### 2.5 Extraction and analysis of secondary metabolites

The bacterial CBS-B5 strain was cultivated overnight in TSB at 30 °C with continuous agitation to obtain a fresh bacterial culture. For metabolite production, 25 g of sterilized rice was transferred into sterile 250 mL Erlenmeyer flasks containing 100 mL of TSB medium and inoculated with 5 mL of the overnight bacterial culture. The cultures were incubated at 30 °C for 14 days. Uninoculated control flasks, containing 25 g of sterilized rice and 100 mL of TSB medium without bacterial inoculation, were prepared under identical conditions. Four independent biological replicates were included for both the inoculated and control treatments. Supernatants were collected from each flask on day 14 and centrifuged to remove particulate matter, filtered through a 0.25 μm membrane filter, and subsequently analyzed by LC-MS. The supernatants composition was analyzed using a Shimadzu system (LCMS-8050 triple quadrupole mass spectrometer, Shimadzu, Tokyo, Japan) equipped with an electrospray ionization (ESI) source. Chromatographic separation was performed on a Shim-pack GIST PFPP column (2.1 × 150 mm, 3 μm) maintained at 40°C. The mobile phases consisted of water (A) and acetonitrile (B), both containing 0.1% formic acid, at a flow rate of 0.25 mL/min. The gradient program was as follows: 0–2.0 min, 0% B; 5.0 min, 25% B; 11.0 min, 35% B; 12.5–16.0 min, 50% B; and 16.01–17.0 min, 95% B, followed by a 1 min hold. The sample was injected using a SIL-40C XS autosampler (Shimadzu, Tokyo, Japan).

### 2.6 Metabolite data processing and statistical analysis

Raw LC-MS metabolite abundance data from four biological replicates per treatment were analyzed using Python (v3.13.11). For each metabolite, the number of detected biological replicates was determined separately for the control and CBS-B5 samples. To ensure robust metabolite detection, only metabolites detected in at least three of the four biological replicates within a treatment were considered consistently present. Conversely, metabolites detected in at least three control replicates but absent from all CBS-B5 replicates were classified as unique to the control. For metabolites consistently detected in both treatments, the mean abundance and standard deviation (SD) were calculated using only the detected replicate values. Differences in metabolite abundance between treatments were evaluated using Welch's two-sample *t*-test, which does not assume equal variances between groups. Fold change (FC) was calculated as the ratio of the mean abundance in CBS-B5 samples to that in the control. Metabolites were considered significantly increased when they exhibited an FC ≥ 1.5 with a *P*-value < 0.05, whereas metabolites with an FC ≤ 0.67 and a *P*-value < 0.05 were considered significantly decreased. The final dataset included only metabolites classified as unique to CBS-B5, unique to the control, increased, or decreased. Metabolites that did not satisfy these criteria were excluded from the differential metabolite analysis.

### 2.7 Genomic DNA preparation and PCR fingerprinting

The CBS-B5 bacterial colony was loop-inoculated into sterile Luria–Bertani (LB) broth (Miller formulation; L2542, Sigma-Aldrich, St. Louis, MO, USA) and incubated overnight at 32 °C. A 5 mL aliquot of the overnight culture was used for total genomic DNA extraction using the QIAamp^®^ DNA Mini Kit on the QIAcube Connect automated DNA extractor, according to the manufacturer's instructions. After extraction, the quality and quantity of the extracted DNA were assessed using a NanoDrop™ spectrophotometer and a Qubit™ fluorometer (Thermo Fisher Scientific, Waltham, MA, USA), respectively. Genomic DNA was stored at −20 °C for further analysis.

PCR amplification was carried out using Platinum Direct PCR Universal Master Mix (Invitrogen, A44647500) in a final reaction volume of 20 μL containing genomic DNA (approximately 50 ng/μL) and 1 μM of each primer. Genotypic fingerprinting of CBS-B5 was performed using repetitive element–based PCR fingerprinting, including BOX, ERIC, and GTG5-PCR assays. The primers used were ERIC-f (5′-ATGTAAGCTCCTGGGGATTCAC-3′) and ERIC-r (5′-AAGTAAGTGACTGGGGTGAGCG-3′) for ERIC-PCR (Versalovic et al., 1991), GTG-f (5′-GTGGTGGTGGTGGTG-3′) for GTG5-PCR (Švec et al., 2008), and BOXA1R-f (5′-CTACGGCAAGGCGACGCTGACG-3′) for BOX-PCR (Dombek et al., 2000). All PCR amplifications were performed under the following conditions: an initial denaturation at 95 °C for 2 min, followed by 5 cycles of denaturation at 95 °C for 30 s, annealing at 53–55 °C for 30 s, and extension at 72 °C for 1 min, with a final extension step at 72 °C for 10 min. PCR products were separated by electrophoresis on 1.5% agarose gels prepared in TBE buffer, stained with SYBR Green I nucleic acid gel stain (Sigma-Aldrich, St. Louis, MO, USA), and visualized using a gel documentation system. The resulting banding patterns and the presence of amplified DNA fragments generated by BOX-, ERIC-, and GTG5-PCR were recorded and used for molecular identification and genotypic characterization of the CBS-B5 strain.

### 2.8 Genome sequencing, assembly, and annotation

Genome sequencing was carried out at Novogene (HK) Company Limited. The sequencing library was prepared for Illumina sequencing and run on a NovaSeq X Plus platform (Illumina, San Diego, CA, USA) to generate 150 bp paired-end reads. Sequencing was performed to achieve high genome coverage based on the expected genome size of approximately ~5.0 Mb, ensuring sufficient depth for accurate assembly and downstream analyses. The raw sequencing reads were subjected to quality control and adapter trimming using Fastp (v0.24.0) with default parameters (Chen et al., 2018).

To obtain high-quality, clean data, the low-quality reads, Illumina adapters, and bases with quality lower than Q30 were filtered. The purity of the sequencing library and the absence of significant exogenous DNA contamination were confirmed using FastQ Screen (version 0.16.0) (Wingett and Andrews, 2018) using default parameters. The screening was performed against the default FastQ Screen database, which includes genomes from *Homo sapiens, Mus musculus*, and other common contaminants, as well as a full representative set of viral, and archaeal genomes. The high-quality, contaminant-free reads were subsequently used for *de novo* genome assembly using Unicycler version 0.5.1 (Wick et al., 2017). Genome assembly was performed using default parameters in normal mode, with a minimum contig length threshold of 1,000 bp. Assembly quality was assessed using QUAST version 5.3.0 (Gurevich et al., 2013) with the same minimum contig length cutoff (1,000 bp). Genome completeness and contamination were estimated using CheckM2 (Chklovski et al., 2023). Genome quality was evaluated according to MIMAG standards, with high-quality genomes defined as having ≥90% completeness and ≤ 5% contamination (Bowers et al., 2017). The assembled contigs were screened for putative plasmid-derived contigs using the mob_suite software v3.1.9 (Robertson and Nash, 2018). The assembled genome was annotated using the NCBI Prokaryotic Genome Annotation Pipeline (PGAP; v6.10) (Tatusova et al., 2016) and the genomic features visualized using the Proksee web-server (Grant et al., 2023).

### 2.9 Genome-based taxonomic classification and phylogenomic analysis

The assembled genome of the CBS-B5 strain was taxonomically classified using GTDB-Tk v2.6.1 (Chaumeil et al., 2022) and FastANI v1.34 (Jain et al., 2018). GTDB-Tk was used for standardized genome-based classification and to obtain average nucleotide identity (ANI) and alignment fraction (AF) values relative to GTDB reference genomes. GTDB-Tk was executed using the default parameters. Species-level relatedness was further assessed using FastANI against 67,761 curated bacterial genomes retrieved from the NCBI database (accessed in November 2025), including only genomes annotated by NCBI RefSeq or GenBank submitters as chromosome-level or complete genome assemblies, excluding atypical and metagenome-assembled genomes.

For phylogenomic analysis, proteome-level comparisons were performed using OrthoFinder v3.0.1b1 (Emms and Kelly, 2019) on 50 closely related genomes (top 25 identified independently by GTDB-Tk and FastANI). OrthoFinder was run using multiple threads and the multiple sequence alignment (MSA) mode to identify orthogroups and extract single-copy orthologs across all included proteomes. These single-copy core genes were concatenated into a genome-wide alignment without manual intervention. The OrthoFinder command used was (orthofinder -f proteome-dir -t 56 -a 56). A maximum-likelihood phylogenomic tree was inferred using IQ-TREE v2.4.0 (Minh et al., 2020) with automatic model selection. Branch support was evaluated using 1,000 ultrafast bootstrap replicates and 1,000 SH-aLRT tests. The resulting phylogenomic tree was visualized and annotated using the Interactive Tree of Life (iTOL) platform (Letunic and Bork, 2024). The list of reference genomes used in the phylogenomic analysis is provided in the Supplementary Table S1.

### 2.10 Gene ontology and functional classification

Gene Ontology (GO) annotation was performed to functionally characterize the CBS-B5 genome and support interpretation of phenotypic and metabolomic data. Protein-coding sequences predicted by the NCBI Prokaryotic Genome Annotation Pipeline (PGAP) were annotated using eggNOG-mapper v2.1.1 (Cantalapiedra et al., 2021) in protein mode with DIAMOND searches aligned against the eggNOG v5.0 database, which infers functional annotations based on orthology relationships and conserved protein domains. High-confidence ortholog assignments were retained using a minimum sequence identity threshold of 90% and a minimum query coverage of 90%, with analysis restricted to bacterial taxa. Only non-electronic GO evidence codes were considered. To improve computational efficiency, all computations were performed using 56 CPU threads.

### 2.11 Mobile genetic elements, CRISPR arrays, and associated cas gene

Mobile genetic elements (MGEs) were identified by screening the predicted proteome against mobileOG-db v2.0.1 using mobileOG-pl (kyanite) (Brown et al., 2022). The database comprises 4,153,117 curated hallmark proteins representing major MGE classes, including insertion sequences, integrative and conjugative elements (ICEs), plasmids, and bacteriophages. High-confidence hits were retained using stringent thresholds (*e*-value ≤ 1 × 10^−20^, amino acid identity ≥90%, and query coverage ≥90%), following a previously published study (Da Silva Morais et al., 2024). CRISPR arrays and associated cas genes were identified using CRISPRCasFinder v4.2.20 (Couvin et al., 2018). CRISPR arrays were evaluated based on repeat length, spacer number, and evidence level as defined by the CRISPRCasFinder scoring scheme. Cas genes were identified independently and classified according to CRISPR-Cas system types.

### 2.12 Horizontal gene transfer analysis

Putative horizontally transferred genes were identified using HGTector 2.0b3 (Zhu et al., 2014), which detects putative horizontal gene transfer (HGT) based on taxonomic incongruence between query proteins and their homologs. Proteome sequences predicted from the CBS-B5 genome were searched against a reference database using DIAMOND v2.1.17.171 (Buchfink et al., 2021), and taxonomic assignments were evaluated using the NCBI taxonomy database (Federhen, 2012). HGTector was executed in two steps: similarity search (hgtector search) followed by statistical classification of predicted HGT candidates (hgtector analyze) using default parameters.

### 2.13 Resistome prediction and virulome analysis

The CBS-B5 predicted proteome was screened for antimicrobial resistance (AMR) determinants and associated virulence factors. Resistance Gene Identifier (RGI) v3.2.1 with the comprehensive Antibiotic Resistance Database (CARD) as reference data (v1.1.9) (Alcock et al., 2023). Additional screening for virulence-associated genes was performed using Abricate v1.0.1 (https://github.com/tseemann/abricate) against the Virulence Factor Database (VFDB) (Chen et al., 2016). The analysis was run with stringent parameters to a minimum sequence identity and coverage of 90% to ensure high-confidence matches.

### 2.14 Assessment of AMR and virulence-associated gene mobility potential

The mobility potential of antimicrobial resistance and virulence-associated genes was evaluated based on genomic context and proximity to mobile genetic elements. Genomic coordinates of AMR genes identified using RGI were compared with MGE annotations derived from mobileOG. Physical distances between AMR genes and the nearest MGE were calculated using BEDTools v2.31.1 (Quinlan and Hall, 2010). Distance ranges were applied as qualitative categories to distinguish relative mobilization potential: genes located within ≤ 5 kb of an MGE were considered to have high mobilization potential, those located 5–20 kb away were considered to have moderate potential, and genes located beyond 20 kb were considered to have low or negligible potential. These categories were used as heuristic indicators of relative co-mobilization likelihood, reflecting the localized activity of MGEs rather than absolute biological cutoffs. HGTector results were integrated to distinguish intrinsic from horizontally acquired AMR genes. Virulence-associated genes identified using ABRicate were similarly evaluated based on overlap with HGTector-predicted regions. Genes overlapping putative HGT regions were considered putatively horizontally acquired, whereas non-overlapping genes were interpreted as vertically inherited. Proximity to MGEs was used as contextual support rather than definitive evidence of active mobility.

### 2.15 Secondary metabolites prediction

Biosynthetic gene clusters (BGCs) in CBS-B5 genome were predicted using antiSMASH (version 8.0.1) (Blin et al., 2025). The PGAP-annotated GenBank file was used as input, and the analysis was performed in bacterial mode. To ensure comprehensive detection of secondary metabolite biosynthetic pathways, full HMM-based domain annotation was enabled, and Prodigal was used for gene prediction on contigs lacking annotated coding sequences. Additional options were applied to identify active site features, known cluster similarities, subcluster architectures, and secondary metabolite orthologous groups (smCOGs). The analysis was performed using 56 CPU threads. Predicted clusters were classified by biosynthetic type and similarity to known BGCs.

### 2.16 Genome-based identification of PGPR-associated genes

PGAP-annotated protein-coding genes were screened to evaluate the genomic potential for plant growth-promoting rhizobacteria (PGPR) traits. A curated list of canonical PGPR marker genes was compiled from established literature, including genes associated with phosphate solubilization, siderophore production, and abiotic stress tolerance. PGPR inference was based on a binary gene presence/absence approach, where PGAP annotations were screened for exact matches to the curated marker list (1 = present, 0 = absent). The distribution of PGPR-related genes was visualized using Python (pandas, matplotlib) in a multi-panel layout, with each panel representing a distinct PGPR trait.

### 2.17 Comparative genome analysis

Reference genome sequences of *Bacillus anthracis* (GCF_000008445.1), *Bacillus cereus* (GCF_006094295.1), and *Bacillus thuringiensis* (GCF_000161615.1) were retrieved from the NCBI database (accessed, March-2026). These genomes were compared with the sequenced CBS-B5 strain. All genomes were annotated using Prokka v1.14.6 (Seemann, 2014) to ensure consistency across datasets and to generate standardized GFF3 files. Comparative genome analysis was performed using Panaroo v1.6.0 (Tonkin-Hill et al., 2020) with moderate cleaning parameters to reduce annotation artefacts while preserving true biological variation. Orthologous gene clusters were identified across all genomes, and a gene presence/absence matrix was generated. Based on this matrix, genes were classified into core genes (present in all strains) and strain-specific genes (unique to individual genomes). The numbers of shared and unique gene clusters were calculated directly from the Panaroo output. Visualization of gene distribution was performed using R, including Venn diagrams and UpSet plots derived from binary presence/absence data. All analyses were conducted using default parameters unless otherwise specified.

## 3 Results

### 3.1 Bacterial isolation and morphological characterization

**Figure 1:**
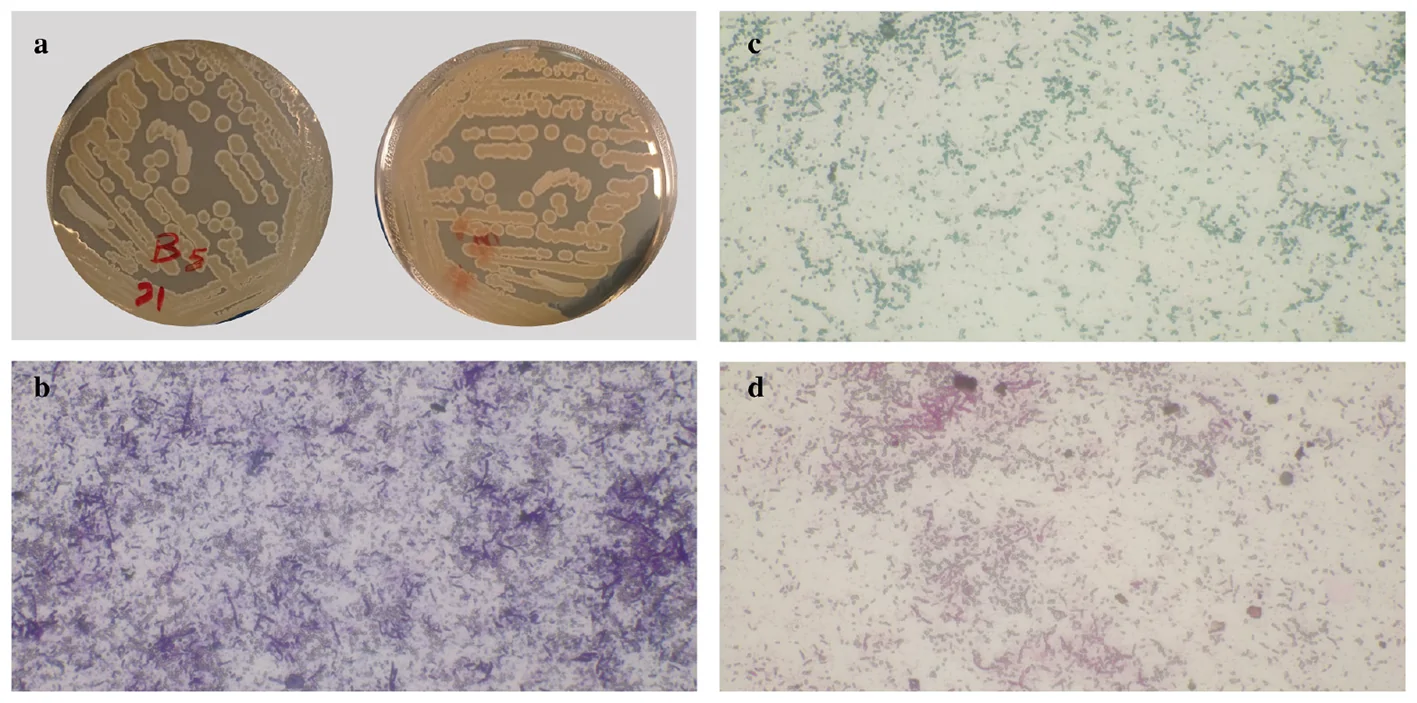
Morphological characterization of the *B. cereus* CBS-B5 strain. **(a)** Macroscopic morphology of the colonies on plate agar. **(b)** Microscopic morphology of Gram-positive cells stained with crystal violet. **(c)** Spores and endospores of strain CBS-B5 stained with malachite green stain appear green/light blue. **(d)** Combined endospore and vegetative cell stain (malachite green and safranin), with endospores appearing green/light blue and vegetative cells appearing pink/red.

The designated strain CBS-B5 was successfully isolated from the rhizosphere soil of sugar beet on nutrient agar. The strain exhibiting morphological characteristics consistent with the *Bacillus* genus was selected for detailed morphological characterization and further analysis. Macroscopically, the CBS-B5 strain formed large, opaque, creamy to light beige colonies with irregular margins and a rough surface after incubation at 30 °C for 24–48 h (Figure 1a). Gram staining revealed that CBS-B5 strain consisted of Gram-positive cells that retained the crystal violet stain and appeared purple under light microscopy (Figure 1b). The cells were rod-shaped and occurred in short chains. Endospore formation in the CBS-B5 strain was confirmed using the malachite green staining method. Endospore staining demonstrated the presence of centrally and subterminally located endospores, which stained green with malachite green, while vegetative cells appeared pink following safranin counterstaining (Figures 1c, d). Overall, the morphological features for the CBS-B5 strain, including cell shape, Gram reaction, and endospore morphology were consistent with standard taxonomic descriptions of *Bacillus cereus* group.

### 3.2 Heat stress recovery and salt tolerance assays

**Figure 2:**
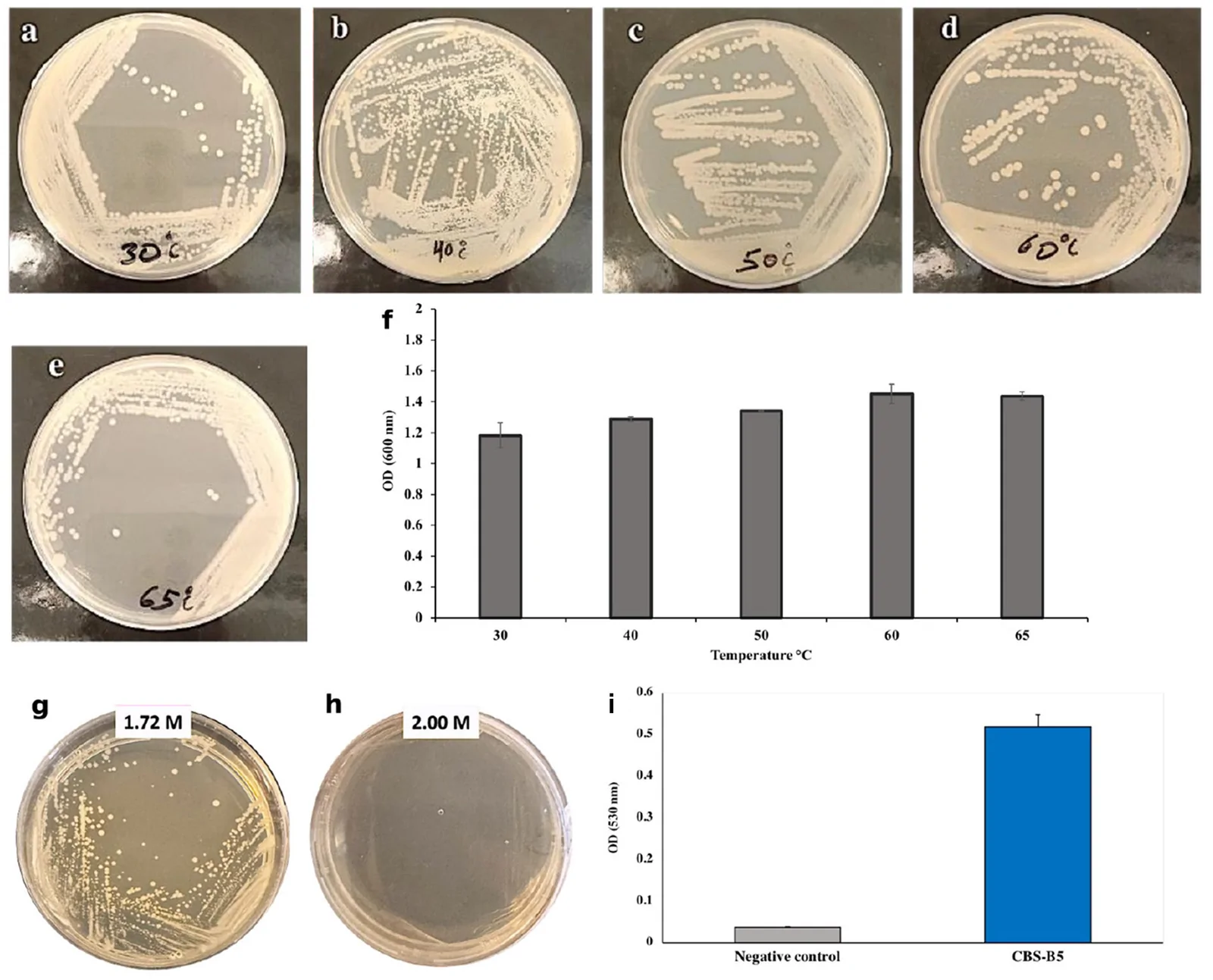
Phenotypic characterization of the *Bacillus cereus* CBS-B5 strain. **(a–e)** Recovery following heat stress after 30 min exposure to different temperatures: **(a)** 30 °C (control), **(b)** 40 °C, **(c)** 50 °C, **(d)** 60 °C, and **(e)** 65 °C, followed by recovery at 30 °C. Visible colony growth after the recovery period indicates the ability of CBS-B5 to resume growth following heat stress. **(f)** Optical density (OD_600_) of cultures after recovery from heat stress. **(g, h)** Qualitative salt tolerance assay showing growth on nutrient agar supplemented with 1.72 M (10%) NaCl **(g)** and the absence of detectable growth at 2.00 M (11.62%) NaCl **(h)**. **(i)** Biofilm formation by CBS-B5 after 48 h of incubation, quantified using the crystal violet microtiter plate assay (OD_530_).

Recovery following heat stress was evaluated under liquid culture conditions by measuring growth recovery following exposure to elevated temperatures, as summarized in Figures 2a–f. CBS-B5 exhibited growth after exposure to 40 °C, 50 °C, 60 °C, and 65 °C, with *OD*_600_ values exceeding those of the 30 °C control (Figure 2f). To confirm cell viability, aliquots from each heat-treated culture were plated on tryptic soy agar and incubated at 30 °C. Visible colonies were observed across all tested temperature conditions (Figures 2a–e).

Salt tolerance of the CBS-B5 bacteria strain was assessed by streaking onto nutrient agar plates supplemented with varying NaCl concentrations ranging from 1% (0.172 M) to 11.62% (2.0 M). Bacterial growth was observed up to 1.72 M (10%) NaCl but was inhibited at 2.00 M NaCl (11.62%) (Figures 2g, h, respectively). These results indicate that strain CBS-B5 exhibited visible growth at NaCl concentrations up to 1.72 M (10%) under the tested conditions, indicating the ability to tolerate elevated salinity in qualitative growth assays (Egamberdieva et al., 2015).

### 3.3 Phosphate solubilization and biofilm formation assays

The phosphate solubilization ability of CBS-B5 was evaluated on Pikovskaya's agar medium containing insoluble calcium phosphate. CBS-B5 exhibited visible growth on Pikovskaya's agar after 7 days of incubation at 32°C. However, no clear halo zones were observed surrounding the colonies, indicating the absence of detectable calcium phosphate solubilization under the tested conditions.

For biofilm-forming ability, CBS-B5 was assessed using the crystal violet microtiter plate assay. The strain exhibited consistently high optical density values (OD_530_≈0.52–0.55) across all three replicates (R1–R3), whereas the negative control showed negligible absorbance (Figure 2i). Based on the calculated OD cut-off (OD_*c*_) and the classification criteria, strain CBS-B5 was categorized as a strong biofilm producer.

### 3.4 Determination of the hemolytic activity and antibiotic susceptibilities

The hemolytic activity of *B. cereus* CBS-B5 was evaluated on 5% blood agar after aerobic incubation at 32 °C for 48 h. Visual inspection of the inoculated plates revealed distinct hemolysis surrounding the bacterial colonies compared with the uninoculated control plate. The CBS-B5 strain produced clear zones around the colonies, indicating complete lysis of red blood cells and corresponding to a β-hemolytic reaction. No greenish discoloration characteristic of α-hemolysis or absence of γ-hemolysis was observed. The control plate showed no hemolytic activity, confirming the specificity of the observed reaction. Based on these results, *B. cereus* CBS-B5 was classified as a β-hemolytic strain (Supplementary Figure S1).

We assessed the antimicrobial susceptibility of CBS-B5 for six antibiotics using the disc diffusion method (Table 1). Disk-diffusion testing showed large inhibition zones for mitomycin (38.8 mm), chloramphenicol (31.5 mm), tetracycline (25.7 mm), and ceftazidime (22.5 mm), while azithromycin and penicillin produced zones of 19.1 mm and 5.5 mm, respectively. Consequently, the strain exhibited sensitivity to most antibiotics tested. The CBS-B5 strain was susceptible (S) to ceftazidime, chloramphenicol, tetracycline, and mitomycin; intermediate (I) to azithromycin; and resistant (R) to penicillin (Supplementary Figure S2).

**Table 1:** Antibiotic susceptibility profile of CBS-B5.

Antibiotic (class)	Associated resistance genes	*In vitro* result
Ceftazidime (β-lactam/3^rd^-gen cephalosporin)	*bla, bla2*	S (22.5)
Chloramphenicol	*catA*	S (31.5)
Tetracycline	Putative *tet* (e.g., *tetL/M/45*)	S (25.7)
Mitomycin C	*satA*	S (38.8)
Penicillin (β-lactam)	*bla, bla2*	R (5.5)
Azithromycin (macrolide)	*mphK, mphL*	I (19.1)

Values are inhibition-zone diameters (mm). Phenotypes: R, resistant; I, intermediate; S, susceptible (Clinical and Laboratory Standards Institute (CLSI), 2016).

### 3.5 Genotypic characterization using PCR fingerprinting

Genotypic characterization of CBS-B5 was conducted using repetitive element-based PCR fingerprinting, including BOX, ERIC, and GTG5-PCR assays. Each primer system generated multiple DNA fragments of different sizes and unique banding patterns for strain CBS-B5, reflecting a specific genomic fingerprint. The combined fingerprinting profiles showed clear amplification patterns with well-defined bands, supporting accurate genotypic characterization of the strain (Supplementary Figure S3).

### 3.6 LC-MS-based profiling of secondary metabolites

LC-MS analysis identified a diverse set of metabolites after 14 days of CBS-B5 incubation with rice seeds, which were used as a nutrient substrate under laboratory conditions. Differential metabolite analysis was performed using a stringent filtering strategy requiring a metabolite to be detected in at least three of the four biological replicates within each treatment. Metabolites were subsequently classified as unique to a treatment or as differentially abundant based on fold change and statistical significance (Table 2). Two metabolites were detected exclusively in CBS-B5 samples, namely *3-dehydroquinic acid* and *asparagine*, both of which were absent from all control replicates. In contrast, *cysteine* and *guanosine monophosphate* were detected only in the control samples and were not observed in CBS-B5.

**Table 2:** Differential metabolites detected in rice seed cultures inoculated with CBS-B5 compared with uninoculated rice seed controls.

Metabolite	Fold change	*P*-value	Classification
3-Dehydroquinic acid	NA	NA	Unique to CBS-B5
Asparagine	NA	NA	Unique to CBS-B5
Cysteine	NA	NA	Unique to control
Guanosine monophosphate	NA	NA	Unique to control
Choline	2.467	0.001	Increased
Citric acid	2.136	0.022	Increased
Guanine	3.832	0.007	Increased
Hypoxanthine	2.493	0.008	Increased
Inosine	2.204	0.019	Increased
Isocitric acid	2.141	0.020	Increased
Phenyllactic acid	6.984	0.016	Increased
Cytidine monophosphate	0.205	0.048	Decreased
Pantothenic acid	0.241	0.022	Decreased
Threonine	0.420	0.017	Decreased

Metabolites were classified as unique to a treatment or differentially abundant based on detection in at least three of four biological replicates, a fold change (FC) threshold of ≥1.5 or ≤ 0.67, and Welch's *t*-test (*P* < 0.05).

Among the metabolites consistently detected in both treatments, seven showed significantly higher abundance following CBS-B5 inoculation (FC ≥ 1.5, *P* < 0.05). These included *choline, citric acid, guanine, hypoxanthine, inosine, isocitric acid*, and *phenyllactic acid*. Phenyllactic acid exhibited the largest increase, with an approximately sevenfold higher abundance in CBS-B5 samples (FC = 6.98, *P* = 0.016). Citric acid and isocitric acid were also significantly enriched, showing fold changes of 2.14 and 2.14, respectively, indicating an enhancement of organic acid metabolism following bacterial treatment. Conversely, three metabolites were significantly reduced in CBS-B5 samples relative to the control (FC ≤ 0.67, *P* < 0.05), including *cytidine monophosphate, pantothenic acid*, and *threonine*. These metabolites exhibited fold changes ranging from 0.21 to 0.42, indicating a marked reduction in their abundance after CBS-B5 inoculation (Table 2).

### 3.7 Genome sequencing, assembly, and annotation

High-throughput sequencing of the CBS-B5 genome generated a total of 19.35 million paired-end reads, corresponding to 2.90 Gbp of raw data. Initial quality assessment showed high base quality, with 96.53% of bases above Q30 and a GC content of 37.26%. After quality filtering and adapter trimming using Fastp, 99.73% of reads (19.30 million) were retained, with a slight increase in Q30 bases to 96.65% and a GC content of 37.12%. Only a small fraction of reads was removed due to low quality (0.0001%), excessive ambiguous bases (0.01%), or short length (0.25%), indicating overall high sequencing quality and minimal data loss. Contamination screening using FastQ Screen revealed that the majority of reads were unmapped to non-target genomes prior to filtering, with only a negligible proportion showing minor matches to human, mouse, fungal, or vector sequences. Following filtering, all reads were completely unmapped to the reference contaminant databases, confirming the absence of detectable exogenous DNA contamination. These results indicate high sequencing quality, low contamination levels, and sufficient depth for downstream genome assembly.

High-quality sequences of CBS-B5 were *de novo* assembled using Unicycler (Wick et al., 2017), yielding 55 contigs (≥1 kb) with a total assembly size of 5,026,553 bp (~5.02 Mbp). The assembly exhibited an average read depth of 438 × and a GC content of 35%. The genome assembly, annotation, and raw sequencing data have been deposited in the DDBJ/ENA/GenBank databases under accession number JBPRPQ000000000, BioProject PRJNA1280512, BioSample SAMN49845414, and SRA SRR34424015.

**Figure 3:**
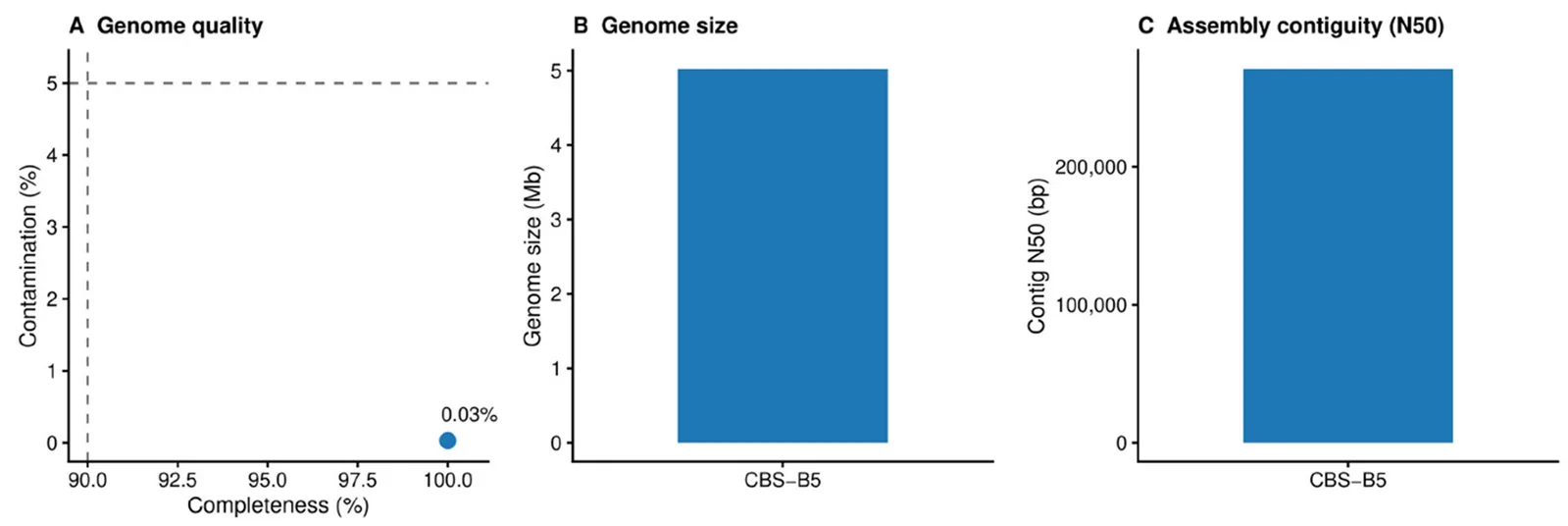
Illustrates the assessment of genome assembly completeness for the CBS-B5. **(A)** Genome completeness and contamination estimated using CheckM2. Dashed lines indicate MIMAG thresholds for high-quality genomes (≥90% completeness and ≤ 5% contamination). The CBS-B5 genome shows extremely low contamination (0.03%). **(B)** Genome size. **(C)** Assembly contiguity represented by contig N50.

Quality assessment of the assembled genome using QUAST (Gurevich et al., 2013) showed that the largest contig was 961,615 bp. The assembly exhibited an N50 of 271,007 bp and an N90 of 53,931 bp, with L50 and L90 values of 5 and 24 contigs, respectively. No ambiguous bases were detected (N's per 100 kbp = 0.0). All 55 contigs exceeded 1,000 bp, and 4.54 Mbp (90.5%) of the total assembly length was contained within 24 contigs of at least 50,000 bp (Supplementary Figure S4). Genome completeness was assessed using the CheckM2 tool (Chklovski et al., 2023), which indicated a completeness of 100% and a contamination level of 0.03% (Figure 3). Screening for plasmid-derived contigs using mob_suite (Robertson and Nash, 2018) did not identify any plasmid-associated sequences in the assembly.

**Figure 4:**
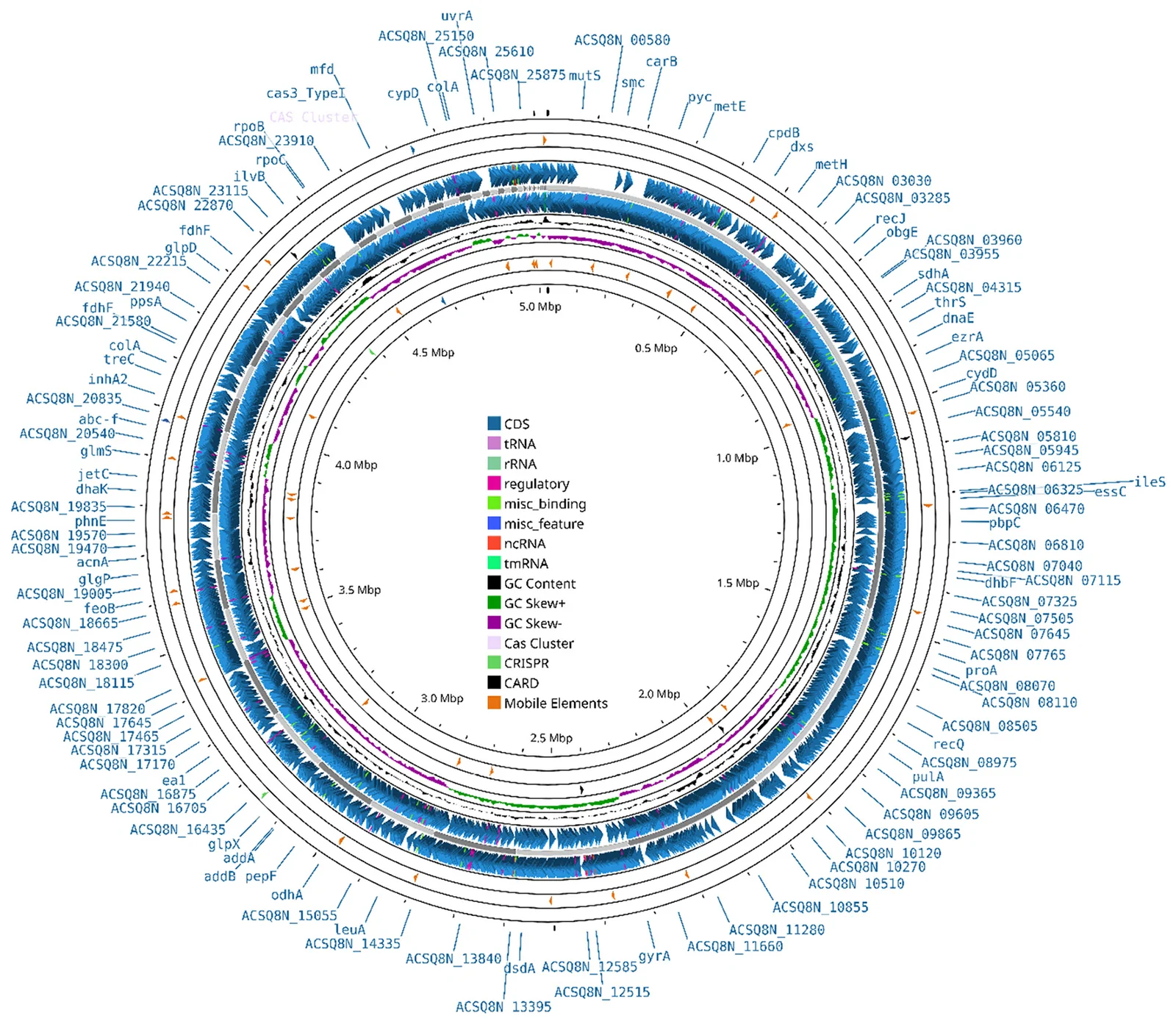
The circular map depicts the CBS-B5 annotated genome feature. From outer to inner, tracks depict CRISPR arrays and cas genes, mobile genetic elements (forward strand), CARD-annotated antimicrobial resistance genes (forward strand), forward-strand CDSs, contig boundaries, reverse-strand CDSs, GC content, GC skew, CARD annotations (reverse strand), mobile genetic elements (reverse strand), and CRISPR arrays and cas genes (reverse strand).

The genome sequence was annotated using the PGAP (Tatusova et al., 2016), which identified a total of 5,234 genes, corresponding to a coding density of 85.4%. Among these, 5,180 coding sequences (CDSs) were detected, including 5,013 protein-coding genes and 167 pseudogenes. In addition, 54 RNA genes were identified, comprising 46 tRNA genes, 5 ncRNAs, and 3 rRNA genes. Key genomic features and assembly statistics of CBS-B5 are summarized in Table 3. A circular representation of the genome generated using Proksee is shown in Figure 4.

**Table 3:** Genome features, assembly completeness, and annotation summary of the CBS-B5 genome.

Genome features		Assembly completeness		Annotation summary (PGAP)	
Feature	Value	Metric	Value	Feature	Value
Number of scaffolds	55	Completeness	100%	Genes (total)	5,234
Number of contigs	55	Contamination	0.03%	CDSs (total)	5,180
Total length	5,026,553 bp	GC content	35%	Genes (coding)	5,013
Percent gaps	0	Average gene length	278.85 bp	CDSs (with protein)	5,013
Scaffold N50	271 kbp	Max contig length	961.6 kbp	Genes (RNA)	54
Contig N50	271 kbp	Coding density	85.4%	rRNAs (16S, 23S)	3
				tRNAs	46
				ncRNAs	5
				Pseudogenes	167

### 3.8 Genome-based taxonomic classification and phylogenomic analysis

GTDB-Tk analysis classified strain CBS-B5 within the *Bacillus cereus* sensu lato group, with average nucleotide identity (ANI) values ranging from 83.47% to 97.7% and alignment fraction (AF) values ranging from 15.7% to 88% compared to 54 closely related reference genomes (Supplementary Table S2). To further assess species-level relatedness, FastANI analysis was performed against 67,761 bacterial genomes. A total of 613 closely related genomes belonging to the *Bacillus* genus were identified, with ANI values ranging from 90.0% to 97.9% and AF values ranging from 75.4% to 93.7% (Supplementary Table S3). The highest similarities were observed with members of the *B. cereus* group, including *Bacillus anthracis, Bacillus cereus*, and *Bacillus thuringiensis*, with ANI values exceeding 97.7% and alignment fractions greater than 92%.

**Figure 5:**
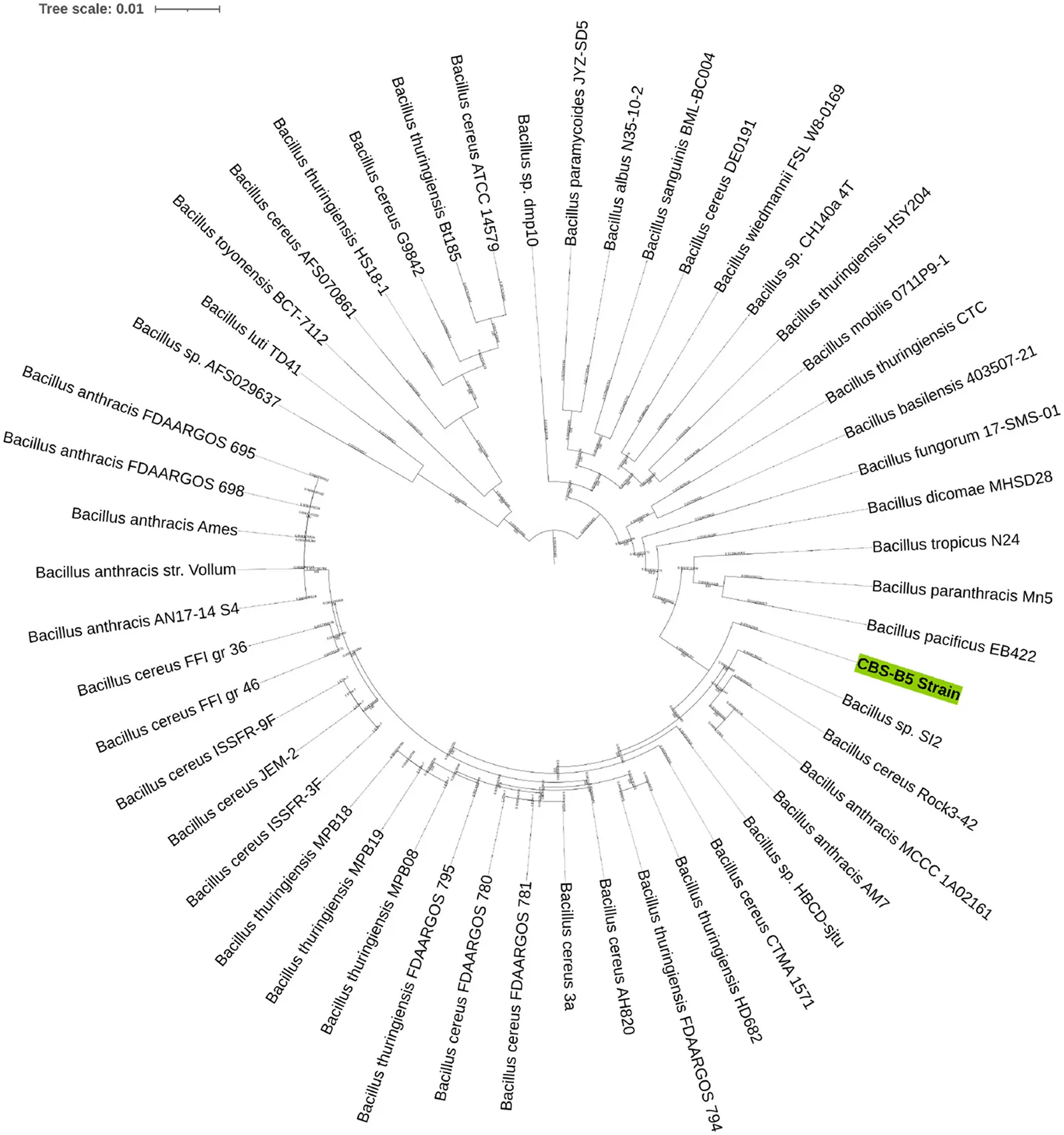
Maximum-likelihood phylogenomic tree reconstructed from concatenated single-copy orthologs identified by OrthoFinder. Branch support values were calculated using 1,000 ultrafast bootstrap and 1,000 SH-aLRT replicates. Strain CBS-B5 (highlighted) clusters within the *Bacillus cereus* sensu lato clade.

Proteome-based phylogenomic analysis was performed using OrthoFinder on a dataset of 50 closely related genomes, comprising the top 25 genomes identified independently by GTDB-Tk and FastANI (Figure 5). A total of 272,273 genes (98.7%) were assigned to 8,598 orthogroups. Half of the genes were contained in orthogroups with 51 or more genes (G50 = 51), distributed across the largest 2,728 orthogroups (O50 = 2,728). A total of 2,704 orthogroups were shared among all analyzed genomes, of which 2,351 consisted of single-copy genes. A maximum-likelihood phylogenomic tree was reconstructed from the concatenated alignment of single-copy orthologs using IQ-TREE with automatic model selection. Branch support was evaluated using 1,000 ultrafast bootstrap replicates and 1,000 SH-aLRT tests. The resulting tree placed strain CBS-B5 within the *Bacillus cereus* sensu lato clade, clustering most closely with *Bacillus cereus* Rock3-42 and *Bacillus* sp. SI2, with strong support values (≥95%).

### 3.9 Gene ontology analysis and functional annotation

**Figure 6:**
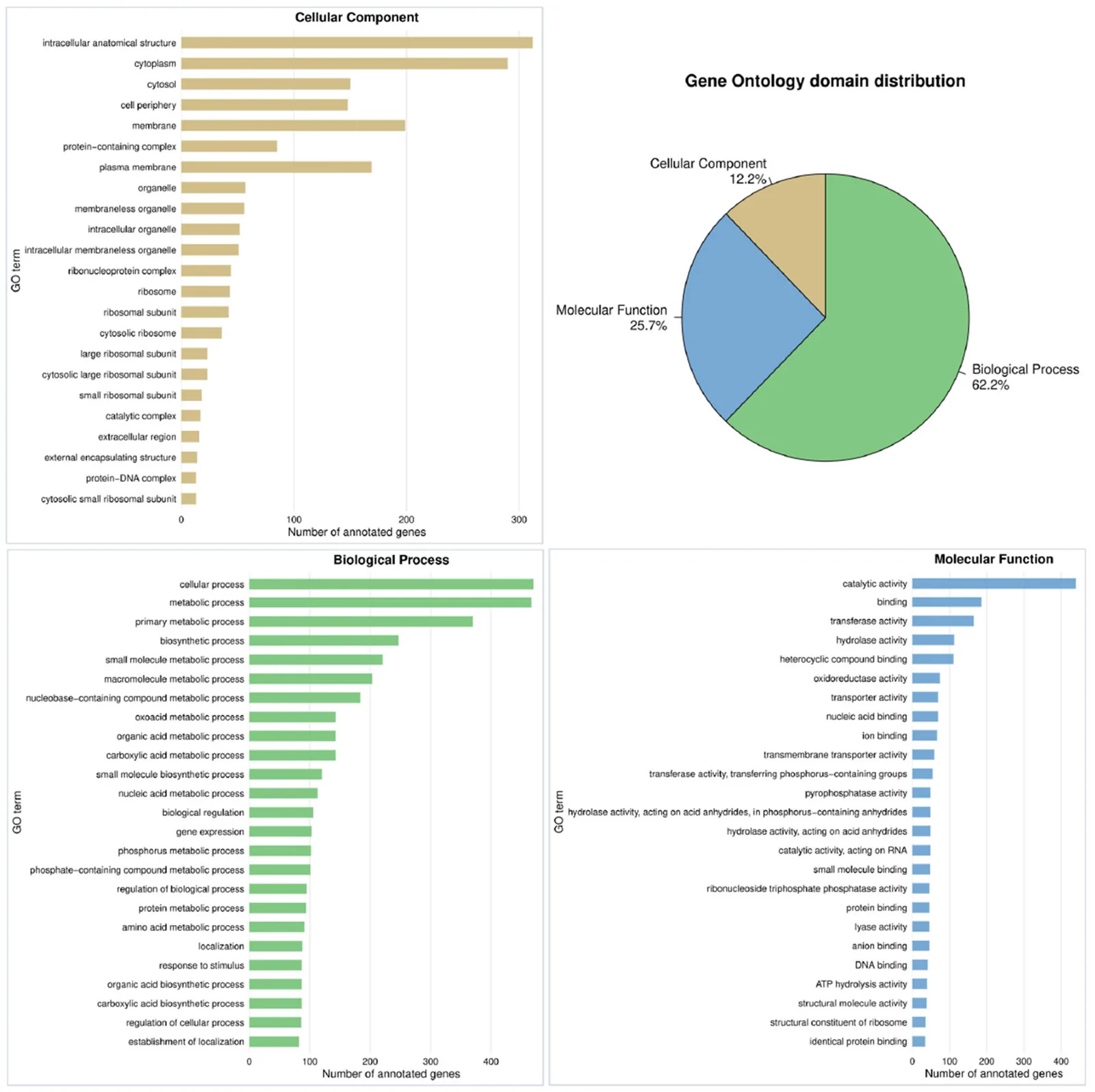
Functional annotation and GO term enrichment of the *B. cereus* CBS-B5 genome. The chart shows the distribution of predicted protein-coding genes across the three main GO categories: biological process, cellular component, and molecular function. For each category, the top 25 GO terms are shown. The bars indicate the number of annotated genes assigned to each term, providing an overview of the genome's functional potential and the primary biological systems it encodes.

Gene Ontology (GO) annotation of the CBS-B5 proteome provided a comprehensive overview of its functional composition across the three main GO domains: biological process, molecular function, and cellular component. Of the 5,013 predicted protein sequences, 4,134 were successfully annotated based on orthologous groups from the EggNOG database. GO classification revealed that biological process terms were predominant (62.2%), followed by molecular function (25.7%) and cellular component (12.2%) (Figure 6). Within the biological process category, the most represented terms were associated with metabolic and cellular processes, including primary metabolism, biosynthetic pathways, and cellular organization. Molecular function annotations were dominated by catalytic activity and binding functions, including transferase, hydrolase, oxidoreductase, and transporter activities. Cellular component annotations primarily corresponded to intracellular structures, including cytoplasmic, membrane-associated, and ribosomal components.

In the biological process domain, 19 functional pathways were identified. Broad categories such as cellular processes (~1,400 genes) and metabolic processes (~1,300 genes) were the most abundant, followed by organic substance metabolic processes (~1,100 genes). In the cellular component category, the majority of genes were associated with intracellular structures, with over 1,200 genes assigned to cell-related terms, followed by cell part (~800 genes) and intracellular anatomical structure (~600 genes). Membrane-associated components, including cytoplasm (~300 genes), membrane (~250 genes), and plasma membrane (~200 genes), were also well represented. Within the molecular function domain, catalytic activity was the most abundant category, encompassing more than 1,600 genes. Binding-related functions were also highly represented, including general binding (~1,200 genes), organic cyclic compound binding (~1,000 genes), heterocyclic compound binding (~800 genes), and ion binding (~600 genes). Additional functional categories included nucleotide binding, small molecule binding, and enzymatic activities such as hydrolase and transferase functions.

### 3.10 Mobile genetic elements, CRISPR arrays, and associated cas genes

MobileOG analysis identified 48 proteins associated with mobile genetic elements (MGEs), including integrases, transposases, recombinases, and conjugation-related proteins. These proteins were classified into functional categories comprising 17 transfer, 25 integration/excision, four phage-related, one replication/recombination/repair, and 1 stability/defense proteins (Supplementary Table S4; Figure 4). CRISPRCasFinder analysis detected two low-confidence putative CRISPR arrays located on contigs 9 and 21, with repeat lengths of 27 and 24 bp, respectively. Each array contained a single spacer and was assigned an evidence level of 1 (Supplementary Table S5; Figure 4). In addition, two *cas* genes were identified on contigs 15, 26, and 28, including two *cas3* genes and one *cas2* gene (Supplementary Table S6; Figure 4). Because the CRISPR arrays were supported only by evidence level 1 and no complete CRISPR-Cas locus was identified, these findings should be interpreted cautiously and may represent degraded or remnant CRISPR elements rather than an active CRISPR-Cas system.

### 3.11 Horizontal gene transfer analysis

HGTector analysis identified 812 genes (16.2% of the studied proteome) predicted to be putatively horizontally transferred in the CBS-B5 genome (Supplementary Table S7). These genes were associated with diverse functional categories, including metabolic enzymes, transporters, transcriptional regulators, and stress response proteins. Representative functions included carbohydrate metabolism (e.g., *gpmA, nagA, nagB*), redox and detoxification processes (e.g., *alkA, adaA*), and transcriptional regulation (e.g., Crp/Fnr, GntR, and MarR family proteins). Taxonomic assignment of best-hit homologs showed that most putatively HGT-associated genes were closely related to sequences from Firmicutes, particularly *Bacillus* species. A smaller subset of genes displayed similarity to more distantly related bacterial taxa. These predictions should be interpreted cautiously, as they are based on taxonomic incongruence and may also reflect conserved ancestry among closely related lineages rather than recent horizontal gene transfer. Among antimicrobial resistance genes, only the *BcII* (*bla2*) gene was predicted as putatively horizontally acquired by HGTector, whereas other resistance determinants did not show HGT signatures.

### 3.12 Resistome prediction and virulome analysis

A comprehensive screening of the *B. cereus* CBS-B5 proteome using the Resistance Gene Identifier revealed multiple AMR genes (Table 4). Four key AMR genes were identified in the CBS-B5 genome, including *fosB* (90.58% identity; fosfomycin resistance), *mphL* (88.26%; macrolide resistance), *BLA1* (94.16%; class A β-lactamase), and *BcII* (92.68%; subclass B1 metallo-β-lactamase; Supplementary Table S8).

**Table 4:** Key antimicrobial resistance genes identified in *B. cereus* CBS-B5 genome.

Gene	Product/function	% identity	Phenotype (AST)
*FosB*	Fosfomycin resistance bacillithiol transferase	90.58	Fosfomycin resistance
*MphL*	Macrolide 2'-phosphotransferase *MphL*	88.26	Macrolides resistance
*BcII*	Suclass B1 metallo-β-lactamase	92.68	Beta-lactams resistance
*BLA1*	Class A β-lactamase	94.16	Beta-lactams resistance (Penicillins, Cephalosporins)

Additional screening for virulence-associated genes was performed using ABRicate and the VFDB database. Virulence profiling identified multiple virulence-associated genes with high sequence identity (>92%; Table 5). Identified determinants included non-hemolytic enterotoxin (*nheA, nheB, nheC, cytK*), immune inhibitor metalloproteases (*inhA*), and the thiol-activated cytolysin *BAS3109 (ALO)*. In total, four AMR-related genes and six virulence-associated genes were detected in the genome.

**Table 5:** Virulence-associated genes and their functions identified in *B. cereus* CBS-B5.

Gene	Protein	% identity	Phenotype
*BAS3109/ALO*	Thiol-activated cytolysin	97.47	Produces a cytolytic effect on host cells, causing cell damage or death, and contributing to bacterial virulence (Chiaverini et al., 2020)
*NheA*	Non-hemolytic enterotoxin A	95.69	Contributes to the cytotoxicity of the Nhe complex, which causes the diarrheal food poisoning syndrome in *Bacillus cereus* (Heilkenbrinker et al., 2013)
*NheB*	Non-hemolytic enterotoxin B	95.37	Essential for the binding of the Nhe complex to host cells, enabling the Nhe toxin to exert its cytotoxic effects and contribute to virulence (Heilkenbrinker et al., 2013)
*NheC*	Non-hemolytic enterotoxin C	92.78	Essential for the assembly and full cytotoxic activity of the Nhe toxin, contributing to virulence (Heilkenbrinker et al., 2013)
*inhA*	Immune inhibitor A	98.75	Contributes to pathogenicity, potentially by degrading host components or interfering with immune responses (Fedhila et al., 2002)
*cytK*	Cytotoxin K	97.03	Forms pores in cell membranes, leading to cell damage, necrosis, and hemolysis. Linked to cases of necrotic enteritis caused by *Bacillus cereus* (Zhao and Sun, 2022)

### 3.13 Assessment of AMR and virulence-associated gene mobility potential

Genomic context analysis revealed that three AMR genes (*fosB, mphL*, and *BcII*) were located >20 kb from the nearest mobile genetic element (MGE), indicating a low relative potential for co-mobilization (Table 6). The *BLA1* gene was positioned ~7 kb from a transposase, suggesting a moderate potential for mobilization based on genomic proximity. HGTector analysis identified only *BcII* as horizontally acquired; however, its distant location from MGEs indicates historical acquisition followed by chromosomal stabilization.

**Table 6:** Integrated assessment of AMR genes based on genomic proximity to MGEs and putative HGT signals in the CBS-B5 genome.

Gene	AMR class	MGE gene	Distance to MGE (bp)	MGE type	HGT	Potential risk
*fosB*	Fosfomycin	*ykyA*	48,980	Transfer	No	Low
*mphL*	Macrolide	*qcrC*	35,206	Transfer	No	Low
*BLA1*	β-lactam	*tnpB*	7,278	Integration/excision	No	Moderate
*BcII*	β-lactam	*TnpB*	48,673	Integration/excision	Yes	Low

In contrast, several virulence-associated genes showed evidence of putative horizontal origin, primarily based on their evolutionary origin rather than distance-based mobility potential. The nheABC operon and *BAS3109* (*ALO*) overlapped with putative HGT-predicted regions, indicating likely putative horizontal acquisition, while *inhA* was located near an putative HGT-enriched locus, suggesting possible co-transfer (Table 7). The *cytK* gene lacked association with both MGEs and putative HGT signals, suggesting vertical inheritance.

**Table 7:** Integrated analysis of virulence-associated genes in the CBS-B5 genome showing their association with putative HGT and MGEs.

Gene	Function	MGE gene	MGE proximity	HGT gene	HGT proximity
*BAS3109*	Virulence-associated protein	*tnp*	12,355	*ALO*	0
*inhA*	Metalloprotease (immune inhibitor A)	qoxD	27,853	*rbsB*	999
*nheA*	Enterotoxin	–	–	ACSQ8N_24750	0
*nheB*	Enterotoxin	–	–	ACSQ8N_24750	1,162
*nheC*	Enterotoxin	–	–	ACSQ8N_24750	2,408
*cytK*	Cytotoxin K	–	–	–	–

Distances are shown in base pairs (bp).

**Figure 7:**
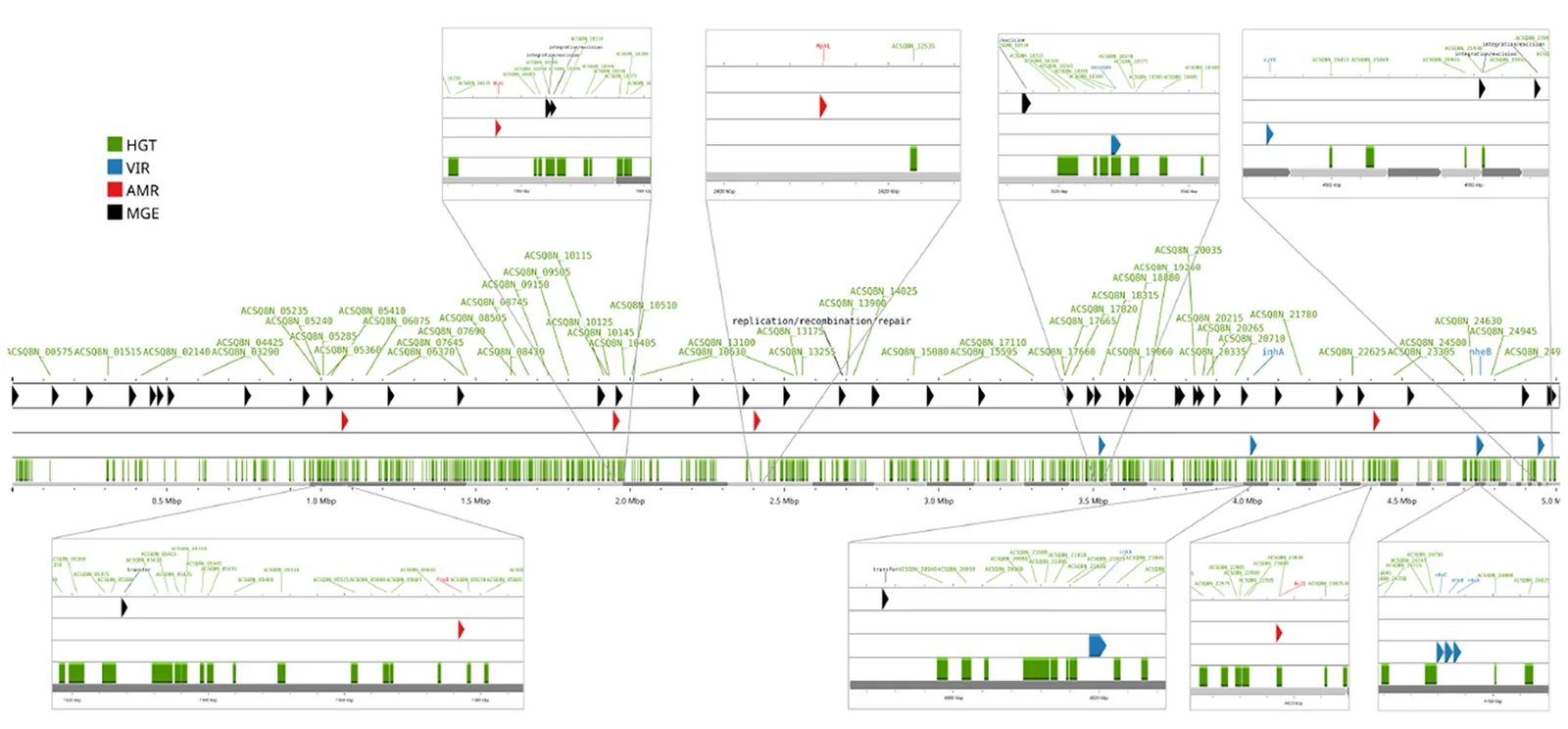
Integrated genome-wide map of predicted putative HGT, MGE, virulence, and AMR genes in the CBS-B5 genome. Chromosomal locations of predicted putative HGT genes (green), virulence genes (blue), AMR genes (red), and MGEs (black) are shown, with zoomed regions illustrating their local genomic context.

Genome-wide mapping and visualization of putative HGT genes, MGEs, virulence-associated genes, and AMR determinants further illustrated these patterns (Figure 7). Genome-wide mapping demonstrated clustering of predicted putative HGT genes in discrete chromosomal regions, with virulence genes co-localizing in some HGT-enriched areas, whereas most AMR genes remained spatially separated from MGEs. Together, these findings indicate putative historical horizontal acquisition of specific virulence loci, with limited evidence for ongoing horizontal dissemination of AMR or virulence genes in the current genomic context for the CBS-B5 strain, suggesting limited potential for ongoing horizontal spread.

### 3.14 Biosynthetic gene cluster prediction and secondary metabolite potential

AntiSMASH analysis identified eight biosynthetic gene clusters (BGCs) distributed across multiple contigs in the CBS-B5 genome (Supplementary Figure S5; Table 8). These included nonribosomal peptide synthetase (NRPS), siderophore-associated gene clusters, RiPP-like, azole-containing RiPP, terpene, and hybrid clusters, indicating a substantial genomic capacity for secondary metabolite production. Two high-confidence siderophore clusters showed strong similarity to the petrobactin and bacillibactin pathways. An NRPS-hybrid cluster displayed low similarity to fengycin-like lipopeptide biosynthesis. Additional RiPP-like and terpene-associated clusters were detected, while one terpene-classified region (Region 17.1) corresponded to molybdenum cofactor biosynthesis rather than a canonical secondary metabolite production. Although several of these clusters showed limited similarity to known biosynthetic pathways, their presence highlights the metabolic diversity of CBS-B5 and suggests potential strain-specific metabolites.

**Table 8:** Summary of biosynthetic gene clusters predicted in the *Bacillus cereus* CBS-B5 genome using antiSMASH.

Region	#Contig	Cluster type	Closest known cluster/similarity
Region 1.1	1	Terpene	No close match detected
Region 2.1	2	Siderophore (NI-type)	Petrobactin biosynthetic cluster (high similarity)
Region 2.2	2	NRPS/NRP-metallophore	Bacillibactin biosynthetic cluster (high similarity)
Region 3.1	3	RiPP-like	No close match detected
Region 3.2	3	Hybrid (NRPS-betalactone-arylpolyene)	Fengycin-like cluster (low similarity)
Region 8.1	8	Azole-containing RiPP	No close match detected
Region 17.1	17	Terpene/other	Molybdenum cofactor biosynthesis (other:other)
Region 38.1	38	NRPS	No close match detected

Importantly, no biosynthetic gene clusters associated with known *Bacillus* food-poisoning toxins or highly cytotoxic secondary metabolites were identified. The predicted BGC repertoire is therefore consistent with an environmental lifestyle and plant-associated functions rather than pathogenicity. Together, these findings indicate that CBS-B5 possesses a diverse but predominantly beneficial secondary metabolite biosynthetic capacity, supporting its classification as a plant growth-promoting bacterium with a limited One Health risk profile (Table 8).

### 3.15 Genomic distribution of canonical PGPR marker genes

**Figure 8:**
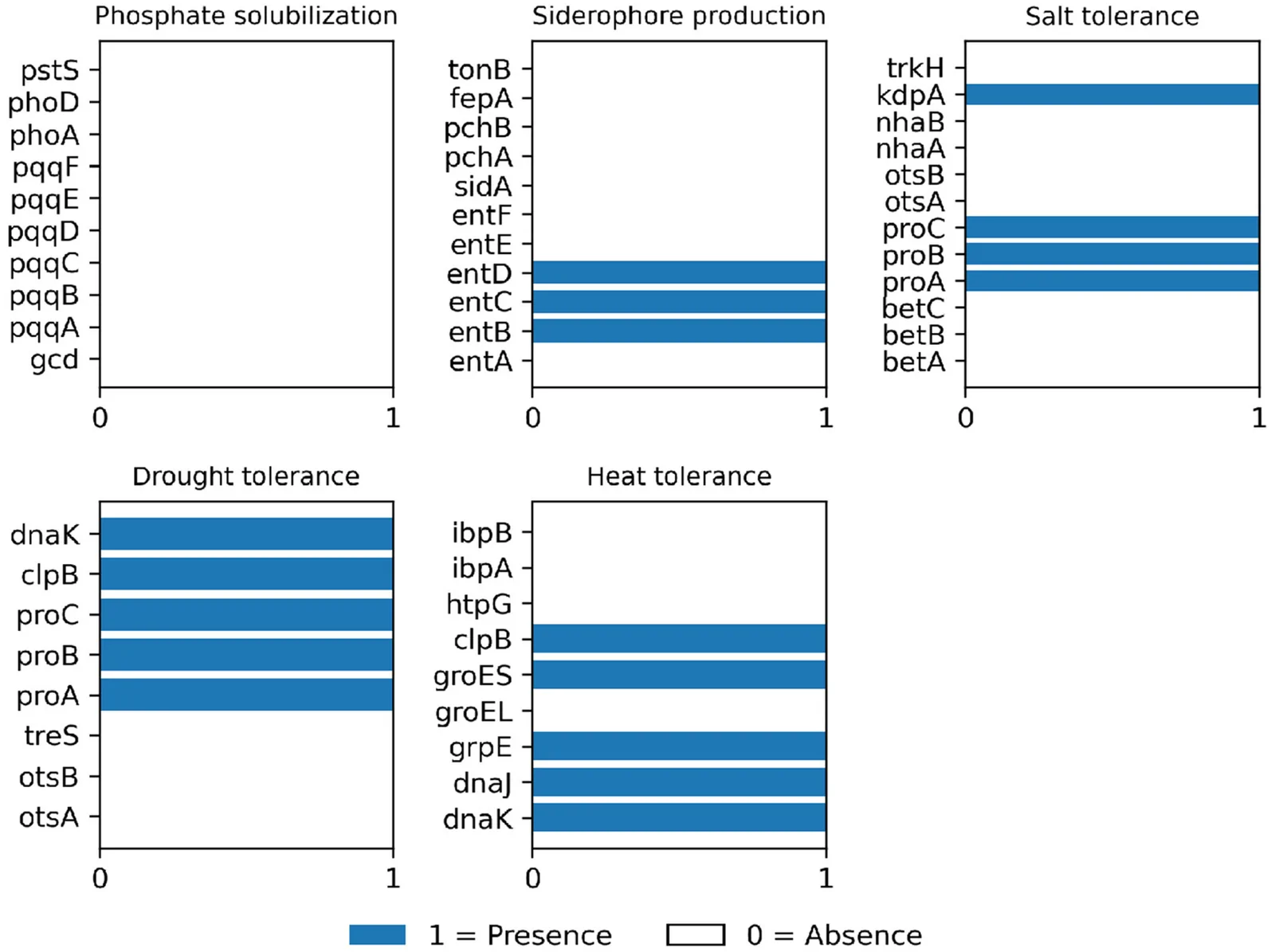
Multi-panel visualization of curated PGPR marker genes showing binary presence (1) or absence (0) across functional trait categories. Each panel represents a single PGPR trait, and only gene-level evidence is displayed.

Genome-based screening revealed a limited but distinct repertoire of canonical PGPR-associated genes (Figure 8). Phosphate-solubilization genes, including *gcd, pqq* operon genes, *phoA, and phoD*, were absent in the genome, suggesting limited genomic potential for phosphate solubilization *via* canonical pathways. In contrast, partial siderophore biosynthesis capacity was indicated by the presence of *entB, entC, and entD*. Moreover, multiple abiotic stress tolerance genes were identified, including proline biosynthesis genes (*proA, proB, proC*) and the potassium transport gene *kdpA*, supporting osmotic stress adaptation. Heat- and drought-related stress response genes (*dnaK, dnaJ, grpE, clpB, groES*) were also present. These findings indicate that the CBS-B5 strain has a well-represented general stress response system.

### 3.16 Comparative genome analysis

**Figure 9:**
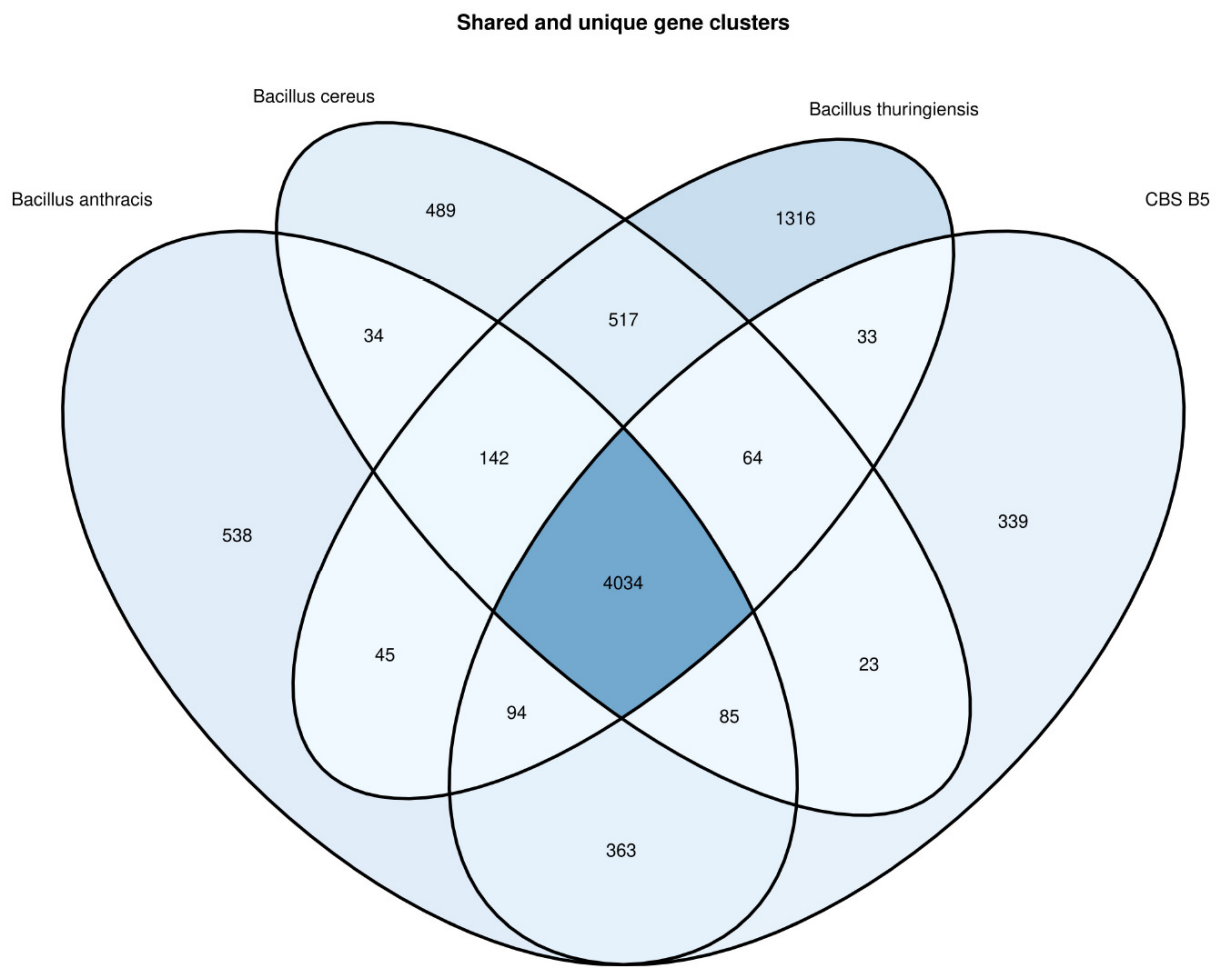
Comparative genome analysis of the *B. cereus* group including strain CBS-B5. Venn diagram showing shared and unique gene clusters among four *Bacillus* genomes. Core and strain-specific gene counts are indicated.

Comparative analysis of the four genomes revealed a total of 4,034 core genes shared among all genomes, representing a substantial conserved genomic backbone within the *Bacillus cereus* group. In addition to the core genome, strain-specific gene content varied considerably among the analyzed genomes. The largest number of unique genes was observed in *Bacillus thuringiensis* (1,316 genes), followed by *Bacillus anthracis* (538 genes) and *Bacillus cereus* (489 genes). Our sequenced strain CBS-B5 contained 339 unique gene clusters, indicating moderate genomic divergence relative to the reference strains (Figure 9; Supplementary Figure S6).

Inspection of strain-specific genes revealed that a large proportion were annotated as hypothetical proteins, reflecting the presence of uncharacterized genetic elements. However, several functionally annotated genes were also identified, including enzymes involved in metabolism, regulatory proteins, transport systems, and mobile genetic elements such as transposases (Supplementary Table S9). Notably, unique genes in *B. cereus* and *B. thuringiensis* included clusters associated with secondary metabolite biosynthesis (e.g., thiocillin and gramicidin-related genes), suggesting differences in ecological adaptation and competitive strategies. In contrast, CBS-B5 specific genes included regulatory proteins, transporters, and stress-related functions, indicating potential niche-specific adaptations.

## 4 Discussion

The *Bacillus cereus* group is well known for its ecological diversity, ranging from beneficial plant-associated bacteria to opportunistic pathogens, making comprehensive evaluation essential for agricultural applications (Stenfors Arnesen et al., 2008; Ceuppens et al., 2013; Ehling-Schulz et al., 2019). Therefore, distinguishing environmentally beneficial strains from clinically relevant ones requires an integrated evaluation combining phenotypic, genomic, and functional analyses.

In the present study, we provide an integrated phenotypic and multi-omics characterization of *B. cereus* CBS-B5, highlighting its ecological adaptability, metabolic versatility, and biosafety profile within a One Health framework. In this context, CBS-B5 exhibits key features of rhizosphere-adapted *Bacillus* strains while maintaining genomic characteristics that limit the risk of pathogenicity or horizontal gene dissemination. In this study, we successfully isolated *Bacillus cereus* strain CBS-B5 from the rhizosphere soil of healthy sugar beet plants using standard microbiological techniques. The morphological and physiological characteristics of CBS-B5 are consistent with standard taxonomic descriptions of the *B. cereus* group, including Gram-positive rod-shaped cells and the ability to form endospores, which contribute to long-term environmental persistence and resilience (Breed et al., 1957; Logan, 2012). Endospore formation is particularly important for survival under fluctuating environmental conditions, including nutrient limitation and abiotic stress (Nicholson et al., 2000).

The phenotypic characterization of *B. cereus* CBS-B5 showed visible growth under elevated salinity conditions, with growth observed up to 1.72 M NaCl (Figure 2g), and recovery following exposure to temperatures as high as 65 °C (Figure 2e). These observations suggest qualitative tolerance to elevated salinity and the capacity to recover from thermal stress under the tested conditions. Such stress-associated traits have been widely reported in plant-associated *Bacillus* spp., particularly those adapted to arid and semi-arid soils, and may contribute to ecological fitness under fluctuating environmental conditions (Vurukonda et al., 2016; Egamberdieva et al., 2017). The ability of *B. cereus* strains to enhance plant tolerance to salinity has been documented previously; for instance, several *B. cereus* strains have been shown to alleviate plant stress caused by heavy metals and salinity (Andy et al., 2023; Jabeen et al., 2022; Sahile et al., 2021; Akhtar et al., 2021; Abou-Aly et al., 2021). Furthermore, the ability of CBS-B5 to recover after thermal stress, as supported by both OD_600_ measurements and colony recovery assays, suggests robust cellular stress-response mechanisms, likely mediated by chaperone systems and osmoprotectant pathways, as reported for other *Bacillus* species (Kim and Schumann, 2009).

The strong biofilm-forming capacity observed in CBS-B5 further supports its enhanced ecological potential. Biofilms play a central role in root colonization, nutrient exchange, microbial persistence, and protection against environmental stressors (Flemming et al., 2016; Beauregard et al., 2013). In *Bacillus* species, biofilm formation has been directly linked to improved plant growth promotion and rhizosphere persistence (Beauregard et al., 2013; Vlamakis et al., 2013). Recent external studies highlight that *B. cereus* strains serve as highly effective bio-inoculants that alleviate abiotic stress in plants through robust biofilm formation, siderophore production, and the regulation of osmolytes (Kulkova et al., 2023; Shi et al., 2025). In contrast, the absence of detectable phosphate solubilization activity under the tested conditions suggests that CBS-B5 does not rely on canonical phosphate-mobilizing pathways. This observation is consistent with previous studies indicating that PGPR can promote plant growth through indirect mechanisms rather than canonical nutrient solubilization (Rodríguez and Fraga, 1999; Sharma et al., 2013). It is possible that CBS-B5 utilizes indirect mechanisms such as microbial interactions to enhance nutrient availability in the rhizosphere.

For genomic characteristics, the whole-genome sequencing and *de novo* assembly of *B. cereus* strain CBS-B5 yielded a high-quality draft genome with 100% completeness and minimal contamination (0.03%), meeting MIMAG standards for high-quality draft genomes. The genome size of approximately 5.02 Mb, distributed across 55 contigs with a GC content of 35% are consistent with other previously reported members of the *B. cereus* group (Rasko et al., 2005; Bazinet, 2017; Liu et al., 2015), supporting the taxonomic placement of CBS-B5 within this lineage. Furthermore, no plasmid-derived contigs were detected, indicating that CBS-B5's genetic determinants are primarily chromosomally encoded, which is a common feature among a certain stable, rhizosphere-associated *B. cereus* strains.

Phylogenomic analysis based on single-copy orthologs placed CBS-B5 within the *B. cereus* sensu lato clade, closely related to *B. cereus* Rock3-42 and *Bacillus* sp. SI2, with strong statistical support (≥95% bootstrap), indicating a close relationship with environmental isolates rather than clinically associated lineages. This placement is consistent with its origin from the rhizosphere and suggests adaptation to soil and plant-associated environments. However, the high genomic similarity (ANI values 97.7%) observed among *B. cereus, B. anthracis*, and *B. thuringiensis* reflects the well-documented taxonomic complexity of this group, which is driven by a conserved core genome and a flexible accessory genome (Helgason et al., 2000; Liu et al., 2015). Therefore, the designation of CBS-B5 as *B. cereus* was not based solely on ANI values but on the combined evidence from GTDB-Tk classification, FastANI analysis, and genome-wide phylogenomic reconstruction using 2,351 single-copy orthologs, which consistently placed CBS-B5 within the *B. cereus* sensu lato clade and in closest association with environmental *B. cereus* isolates.

Furthermore, comparative genome analysis revealed 4,034 core genes shared among CBS-B5, *B. cereus, B. anthracis*, and *B. thuringiensis*, consistent with previous estimates that the *B. cereus* group core genome comprises a substantial conserved fraction (Zeng et al., 2018; Adeleke et al., 2021a). The presence of 339 unique genes in CBS-B5 suggests moderate genomic divergence and potential niche-specific adaptations (Figure 9). Importantly, this pattern is consistent with previous observations that species delineation within the *B. cereus* sensu lato group is often complicated by frequent gene exchange and shared evolutionary history to functional diversity and ecological specialization (Kim et al., 2017; Bazinet, 2017; Koo et al., 2017). The genomic features of CBS-B5, including its close relationship with environmental isolates and the absence of plasmid-encoded virulence determinants, are consistent with an environmental, soil- and plant-associated lifestyle rather than a clinical lineages.

Functional annotation of *B. cereus* CBS-B5 further revealed a genome enriched in metabolic and catalytic activities, consistent with the ecological role of rhizosphere bacteria in nutrient cycling, plant interaction, and environmental adaptation (Mendes et al., 2013). Gene ontology analysis showed a predominance of biological process terms associated with metabolism, biosynthesis, and cellular processes, reflecting the central role of metabolic flexibility in environmental survival (Figure 6). This pattern is characteristic of *Bacillus* species adapted to complex soil ecosystems (Kulkova et al., 2023). Additionally, the enrichment of molecular function categories related to catalytic activity and binding supported by the high representation of transferases, hydrolases, oxidoreductases, and transporter-associated functions aligns with detected metabolites in CBS-B5 related to nitrogen metabolism and stress response, as well as with phenotypic traits such as salt tolerance and biofilm formation, suggesting the capacity of the CBS-B5 strain to utilize a wide range of substrates and respond to environmental fluctuations (Paterson et al., 2017).

At the functional level, CBS-B5 encodes genes associated with oxidative stress response (e.g., *sodA, trxA*) and molecular chaperones and protein folding (*dnaK, dnaJ, grpE, clpB, groES clpB*) align with the strain's tolerance to heat and osmotic stress (Storz and Imlayt, 1999; Nies, 2003; Kulkova et al., 2023; Sivasankari and Anandharaj, 2014; Elsayed et al., 2022).

Genome plasticity was evident through the presence of MGEs and HGTs in the CBS-B5 proteome. The genome of CBS-B5 contains multiple mobile genetic elements associated proteins (48 proteins identified *via* mobileOG-db), including transposases, integrases, recombinases, and conjugation-related proteins. These proteins were classified into functional categories comprising 17 transfer, 25 integration/excision, four phage-related, one replication/recombination/repair, and one stability/defense proteins (Supplementary Table S4). The predominance of integration/excision and transfer-related elements suggests that horizontal gene flow has contributed significantly to genome evolution, as commonly observed in members of the *Bacillus cereus* group (Bazinet, 2017). In addition, HGT is a major driver of bacterial evolution, enabling rapid acquisition of adaptive traits such as antibiotic production, resistance mechanisms, and metabolic flexibility (Soucy et al., 2015; Frost et al., 2005). HGTector analysis suggests that putative horizontal gene transfer may have contributed to the functional diversity of the CBS-B5 genome, with approximately 16.2% of its proteome predicted to be putatively horizontally transferred. Most genes predicted to be putatively horizontally transferred were associated with metabolic pathways (e.g., *gpmA, nagA, nagB*), transport systems, transcriptional regulation (e.g., *Crp/Fnr, GntR*, and *MarR* family proteins), and stress responses (e.g., *alkA, adaA*), consistent with functions involved in environmental adaptation rather than the acquisition of pathogenic traits.

Additionally, the majority of putatively HGT-derived genes were most closely related to sequences from Firmicutes, particularly *Bacillus* species. This pattern is consistent with gene sharing among closely related lineages but may also reflect conserved ancestry rather than recent horizontal gene transfer. Interestingly, the CRISPR-Cas system appears incomplete. CRISPRCasFinder detected only two low-confidence putative CRISPR arrays, each containing a single spacer and assigned an evidence level of 1 (Couvin et al., 2018). In addition, the identified *cas* genes (*cas3* and *cas2*) were dispersed across different contigs and did not form a complete CRISPR-Cas locus. Consequently, these findings should be interpreted cautiously, as they may represent degraded CRISPR remnants or false-positive predictions rather than a functional CRISPR-mediated defense system. Although these observations are consistent with previous interactions between the genome and mobile genetic elements, they do not provide evidence for an active CRISPR-mediated defense system, a pattern that has also been reported in some environmental bacteria (Makarova et al., 2015).

Resistome analyses identified a limited number of AMR genes, including *fosB, mphL, BLA1*, and *BcII* (Table 4). However, genomic context analysis showed that most of these genes are located at substantial distances from MGEs (located >20 kb from the nearest MGE) and lacked HGT signatures, suggesting that they are chromosomally encoded and have a low relative potential for horizontal dissemination (Table 7). Only *BcII* was predicted as putatively horizontally acquired by HGTector. However, this gene was not associated with an MGE, and its distant location from the nearest MGE (48,673 bp) suggests that, if acquired through horizontal gene transfer, it has likely become chromosomally stabilized. The presence of β-lactamase genes *BLA1* and *BclI* is common in *B. cereus* and typically provides intrinsic resistance to penicillins and cephalosporins (Etikala et al., 2022; Dietrich et al., 2021). This is consistent with previous observations that have been reported in environmental *Bacillus* strains, which often harbor intrinsic resistance genes that do not necessarily pose a clinical risk (Martínez et al., 2015).

Phenotypically, CBS-B5 remained susceptible to most tested antibiotics, with resistance observed primarily to penicillin, an intrinsic trait common to the lineage. In this study, phenotypic susceptibility testing was performed using the disc diffusion method according to CLSI guidelines, confirming resistance only to penicillin (zone diameter 5.5 mm), while the strain was susceptible to ceftazidime (22.5 mm), chloramphenicol (31.5 mm), tetracycline (25.7 mm), and mitomycin (38.8 mm), with intermediate susceptibility to azithromycin (19.1 mm; Supplementary Figure S2).

Furthermore, virulome analysis identified multiple virulence-associated genes with high sequence identity (>95%), including the non-hemolytic enterotoxin operon *(nheA, nheB, nheC*), cytotoxin K (*cytK*), immune inhibitor metalloprotease (*inhA*), and the thiol-activated cytolysin BAS3109 (*ALO*) (Table 7). Despite the presence of these genes, CBS-B5 exhibited β-hemolytic activity on blood agar (Supplementary Figure S1), supporting the presence of functional cytolytic factors, which are characteristic of the *B. cereus* group, but such traits are widely distributed among environmental isolates and do not necessarily indicate pathogenicity in agricultural contexts (Ceuppens et al., 2013; Ehling-Schulz et al., 2019). Importantly, the *nhe*ABC operon and *ALO* overlapped with HGT-predicted regions, suggesting a putative horizontal origin. Likewise, *inhA* was located near an HGT-enriched locus (within 999 bp of *rbsB*), which may be consistent with a putative horizontal acquisition. In contrast, the *cytK* gene lacked association with both MGEs and HGT-predicted regions, suggesting vertical inheritance. Similarly, although virulence-associated genes such as *nhe*ABC and *cytK* were detected, their chromosomal localization and lack of association with MGEs suggest a limited potential for horizontal dissemination. However, the predicted HGT associations should be interpreted cautiously, as they are based on computational inference and may also reflect conserved ancestry among closely related Firmicutes rather than recent horizontal gene transfer, which is an important consideration when evaluating the biosafety of microbial strains intended for agricultural applications (Ehling-Schulz et al., 2006).

Genome mining and the biosynthetic gene clusters analysis using antiSMASH revealed that CBS-B5 harbors a diverse repertoire of biosynthetic gene clusters, including siderophore-associated clusters (petrobactin and bacillibactin), NRPS-related pathways, RiPP-like elements, terpenes, and hybrid clusters (Table 8). The identification of petrobactin- and bacillibactin-like siderophore clusters is particularly important, as these molecules facilitate iron acquisition under iron-limited conditions and play a key mechanism of microbial competition, limiting pathogen access to iron in the rhizosphere (Neilands, 1995; Miethke and Marahiel, 2007). The analysis of biosynthetic gene clusters revealed a diverse repertoire, including siderophore, NRPS, RiPP, and terpene clusters. The identification of petrobactin- and bacillibactin-like siderophore clusters is particularly significant, as siderophore production enhances iron acquisition and microbial competitiveness in the rhizosphere (Miethke and Marahiel, 2007). The presence of NRPS and RiPP-like clusters in the CBS-B5 strain suggests their potential production of antimicrobial compounds, which may contribute to biocontrol functions and suppression of plant pathogens (Ongena and Jacques, 2008). Although several predicted clusters showed low similarity to known pathways, these regions may encode strain-specific metabolites that contribute to ecological adaptation. Importantly, no toxin-associated BGCs were detected, further supporting a non-pathogenic, environmentally adapted lifestyle. This observation, together with the broader genomic context, supports a functional profile more consistent with environmental adaptation than pathogenic specialization. The absence of toxin-associated BGCs distinguishes CBS-B5 from emetic strains that harbor the ces gene cluster responsible for cereulide biosynthesis (Ehling-Schulz et al., 2019).

Under laboratory growth conditions, metabolomic analysis provided a preliminary chemical profile of metabolites associated with CBS-B5 growth when rice seeds were used as the nutrient substrate. Because rice seeds served as a nutrient source rather than as a living host, the detected metabolites primarily reflect bacterial metabolism in the presence of plant-derived compounds rather than a true plant-microbe interaction. Among the metabolites detected exclusively in CBS-B5 cultures, 3-dehydroquinic acid was identified as an intermediate of the shikimate pathway, which provides precursors for aromatic amino acids and numerous secondary metabolites (Maeda and Dudareva, 2012). Its exclusive detection following CBS-B5 growth suggests activation of metabolic pathways associated with aromatic compound biosynthesis during utilization of rice seed-derived nutrients. Likewise, asparagine was detected only in CBS-B5 samples. Asparagine functions as an important nitrogen storage and transport compound in plants and microorganisms (Lea et al., 2007), although its accumulation in the present study may simply reflect bacterial metabolism of seed-derived nitrogen rather than active secretion by CBS-B5. Several metabolites showed significantly increased abundance following CBS-B5 growth. The enrichment of citric acid and isocitric acid suggests alterations in tricarboxylic acid (TCA) cycle metabolism during bacterial utilization of the rice seed substrate (Sauer and Eikmanns, 2005). Enhanced accumulation of these organic acids has frequently been associated with active microbial metabolism. Similarly, the marked increase in phenyllactic acid is consistent with previous reports describing its production by diverse bacteria, where it has been associated with antimicrobial activity in several bacterial species (Valerio et al., 2004), although its biological role was not evaluated in the present study.

The increased abundance of guanine, hypoxanthine, and inosine indicates changes in purine metabolism during bacterial growth. These compounds participate in nucleotide turnover and cellular energy metabolism and may reflect enhanced nucleic acid synthesis or degradation associated with active cell proliferation. Likewise, the increase in choline may indicate changes in membrane lipid metabolism or osmotic regulation (Kempf and Bremer, 1998). Conversely, several metabolites decreased following CBS-B5 growth, including cytidine monophosphate, pantothenic acid, and threonine. These reductions likely reflect microbial utilization or transformation of these compounds during growth rather than direct evidence of altered biological pathways. Pantothenic acid is an essential precursor of coenzyme A metabolism (Leonardi et al., 2005). Their reduced abundance therefore suggests consumption during bacterial metabolism of the rice seed substrate, although additional biochemical analyses would be required to confirm the underlying mechanisms. The observed qualitative growth under elevated salinity conditions and the ability to recover following heat stress suggest adaptation to fluctuating environmental conditions typical of soil ecosystems. In addition, the strain exhibited robust biofilm formation, which may enhance surface attachment, persistence, and interactions within the rhizosphere.

Genome-based screening of PGPR markers revealed an incomplete but functionally relevant repertoire. Despite the absence of clear phosphate solubilization activity under the tested conditions, genomic analysis indicated a lack of canonical phosphate-solubilization genes (*gcd, pqq* operon, *phoA, phoD*), suggesting that this function may not be a primary trait of CBS-B5. Instead, the presence of siderophore biosynthesis genes and stress tolerance pathways suggests that CBS-B5 may function as a non-classical PGPR through indirect mechanisms. The predicted siderophore-associated biosynthetic pathways may enhance iron acquisition and limit iron availability to competing microorganisms, thereby improving rhizosphere competitiveness. In addition, the experimentally confirmed ability to form robust biofilms, tolerance to salinity and heat stress recovery, together with the presence of osmoprotection- and stress-response genes, indicates that CBS-B5 may remain metabolically active under adverse environmental conditions where other beneficial microorganisms are less competitive. Collectively, these traits are consistent with indirect plant growth promotion through enhanced rhizosphere persistence, microbial competition, and improved adaptation to environmental stress rather than through direct phosphate solubilization (Glick, 2012; Backer et al., 2018).

From a One Health perspective, the combined genomic, metabolomic, and phenotypic data indicate that CBS-B5 possesses genomic features consistent with a limited potential for horizontal dissemination of antimicrobial resistance and virulence determinants. Specifically, the absence of plasmids, the limited association of AMR genes with MGEs, the lack of toxin-related biosynthetic gene clusters, and the absence of toxin-associated compounds under the tested metabolomic conditions suggest reduced mobility of these genetic determinants. However, the presence of β-hemolytic activity and chromosomally encoded virulence-associated genes, including *nheABC* and *cytK*, represents important biosafety considerations that warrant further experimental evaluation before the strain can be considered for agricultural applications. These findings are consistent with current One Health frameworks, which emphasize the importance of evaluating the genomic context, mobility, and biological significance of resistance and virulence determinants when assessing microbial candidates for agricultural use (Berendonk et al., 2015; Bengtsson-Palme et al., 2018).

Although this study provides a comprehensive multi-omics characterization of *Bacillus cereus* CBS-B5, several limitations should be acknowledged. First, many of the proposed plant growth-promoting traits were inferred from genomic analyses and therefore represent predicted functional potential rather than experimentally validated biological activity. Second, while selected phenotypic traits, including biofilm formation, heat stress recovery, and qualitative growth under elevated salinity conditions, were experimentally evaluated, direct assessment of plant growth promotion and root colonization was not performed. Third, the metabolomic analysis was conducted under laboratory fermentation conditions rather than in a plant-microbe interaction system and therefore reflects the metabolic potential of the strain under the tested conditions. Consequently, future studies should include *in planta* validation, root colonization assays, and field-scale evaluations to confirm the agricultural potential and biosafety of CBS-B5.

## 5 Conclusion

In conclusion, CBS-B5 represents a metabolically versatile and environmentally adapted *Bacillus cereus* strain with genomic and phenotypic characteristics consistent with potential agricultural applications. However, its ability to promote plant growth and colonize plant roots remains to be validated through *in planta* and field-based studies. The observed tolerance to abiotic stress, robust biofilm formation, and production of bioactive metabolites, together with the identified genomic features, suggest the potential for rhizosphere persistence and ecological competitiveness under environmental conditions. Its genomic analyses further indicate a limited potential for horizontal dissemination of antimicrobial resistance and virulence determinants, although additional biosafety evaluation remains necessary. These findings provide a robust multi-omics framework for future validation studies under field conditions. Therefore, future studies should focus on *in planta* validation, root colonization, field-scale evaluation, and interactions with plant microbiomes to fully assess the agricultural potential of CBS-B5 as a biofertilizer or biocontrol agent.

## Data Availability

The complete genome sequence and associated raw sequencing data for *Bacillus cereus* strain CBS-B5 have been deposited in the National Center for Biotechnology Information (NCBI) under the BioProject accession number PRJNA1280512. The BioSample accession is SAMN49845414, and the Sequence Read Archive (SRA) accession is SRR34424015. The assembled whole-genome shotgun project has been assigned the GenBank accession JBPRPQ000000000. Additional supporting data are provided within the Supplementary material accompanying this article.

## Supplementary material

Supplementary Table S1 The top 50 closely related genomes (top 25 identified independently by GTDB-Tk and FastANI) used in phylogenomic analysis.

Supplementary Table S2 The average nucleotide identity and alignment fraction values of CBS-B5 compared to 54 closely related reference genomes using GTDB-Tk.

Supplementary Table S3 The closely related genomes belonging to the *Bacillus* genus identified using FastANI.

Supplementary Table S4 Proteins associated with mobile genetic elements identified using mobileOG analysis.

Supplementary Table S5 The putative CRISPR arrays identified using CRISPRCasFinder.

Supplementary Table S6 The CBS-B5 identified *cas* genes.

Supplementary Table S7 The putatively horizontally transferred genes in the CBS-B5.

Supplementary Table S8 AMR genes identified in the CBS-B5 genome.

Supplementary Table S9 The comparative genome analysis of the CBS-B5 and three genomes within the *Bacillus cereus* group.

Supplementary Figure S1 Hemolytic activity of the *B. cereus* CBS-B5 strain on blood agar. Blood agar plates (5% v/v) after 48 h incubation at 32 °C showing no hemolysis in the control (left) and clear β-hemolytic zones around colonies of *B. cereus* CBS-B5 (right).

Supplementary Figure S2 *In vitro* antibiotic susceptibility testing of *Bacillus cereus* CBS-B5. Disk diffusion assay showing growth inhibition zones around antibiotic-impregnated disks on Mueller–Hinton agar. The abbreviations on the plate correspond to the following antibiotic disks, against which the strain was tested: Cef, ceftazidime (30μ*g*); Chlo, chloramphenicol (30μ*g*); Azi, azithromycin (30μ*g*); Tetr, tetracycline (30μ*g*); Pen, penicillin (30μ*g*); and Mito, mitomycin (30μ*g*). Zones of inhibition indicate the relative susceptibility of *B. cereus* CBS-B5 to the tested antibiotics. The zones of inhibition were measured and interpreted according to CLSI guidelines to determine susceptibility (S), intermediate (I), or resistance (R).

Supplementary Figure S3 Genotypic fingerprinting of *Bacillus cereus* CBS-B5. Agarose gel (1.5%) showing BOX-, ERIC-, and GTG5-PCR banding profiles of strain CBS-B5. PCR products were separated in TBE buffer and visualized after staining with SYBR Green I. The DNA ladder (M) indicates fragment sizes in base pairs (bp).

Supplementary Figure S4 Comprehensive quality assessment of the *B. cereus* CBS-B5 genome assembly. **(a)** Cumulative length plot. The curve shows the cumulative sequence length (Mbp) as contigs are ordered from largest to smallest. The assembly comprises 55 contigs with a total length of 5.02 Mbp. **(b)** GC content distribution. The histogram shows the number of contigs with a given average GC percentage, centered around the genome-wide average of 35.23%. **(c)** Per-contig GC content profile. Lines represent the local GC content (%) across sliding windows for each contig, demonstrating sequence composition homogeneity. **(d)** Nx plot. The curve depicts the minimum contig length (Kbp) needed to cover x% of the genome, from which key metrics like the N50 (271 Kbp) and N90 (53.9 Kbp) are derived, illustrating the high contiguity of the assembly. All analyses were performed on contigs ≥ 1kb.

Supplementary Figure S5 Predicted location of biosynthetic gene clusters in *Bacillus cereus* CBS-B5 genome as identified by antiSMASH tool.

Supplementary Figure S6 UpSet plot showing intersections of gene clusters across genomes, highlighting core and accessory gene distributions.
